# Innate immune regulation in HIV latency models

**DOI:** 10.1186/s12977-022-00599-z

**Published:** 2022-07-08

**Authors:** Rebecca M. Olson, Germán Gornalusse, Leanne S. Whitmore, Dan Newhouse, Jennifer Tisoncik-Go, Elise Smith, Christina Ochsenbauer, Florian Hladik, Michael Gale

**Affiliations:** 1 Center for Innate Immunity and Immune Disease, Department of Immunology, University of Washington School of Medicine, Seattle, WA USA; 2 Vaccine and Infectious Disease Division, Fred Hutchinson Cancer Research Center, Seattle, WA USA; 3 Department of Obstetrics & Gynecology, University of Washington, Seattle, WA USA; 4 Department of Medicine, University of Washington, Seattle, WA USA

**Keywords:** HIV, Latent infection, Reservoir, Innate immunity, Interferon, Interferon-stimulated gene, RIG-I, Immune escape

## Abstract

**Background:**

Innate immunity and type 1 interferon (IFN) defenses are critical for early control of HIV infection within CD4 + T cells. Despite these defenses, some acutely infected cells silence viral transcription to become latently infected and form the HIV reservoir in vivo. Latently infected cells persist through antiretroviral therapy (ART) and are a major barrier to HIV cure. Here, we evaluated innate immunity and IFN responses in multiple T cell models of HIV latency, including established latent cell lines, Jurkat cells latently infected with a reporter virus, and a primary CD4 + T cell model of virologic suppression.

**Results:**

We found that while latently infected T cell lines have functional RNA sensing and IFN signaling pathways, they fail to induce specific interferon-stimulated genes (ISGs) in response to innate immune activation or type 1 IFN treatment. Jurkat cells latently infected with a fluorescent reporter HIV similarly demonstrate attenuated responses to type 1 IFN. Using bulk and single-cell RNA sequencing we applied a functional genomics approach and define ISG expression dynamics in latent HIV infection, including HIV-infected ART-suppressed primary CD4 + T cells.

**Conclusions:**

Our observations indicate that HIV latency and viral suppression each link with cell-intrinsic defects in specific ISG induction. We identify a set of ISGs for consideration as latency restriction factors whose expression and function could possibly mitigate establishing latent HIV infection.

**Supplementary Information:**

The online version contains supplementary material available at 10.1186/s12977-022-00599-z.

## Background

Over 37 million people are currently infected with HIV worldwide but no curative therapy exists [1]. Antiretroviral therapy (ART) effectively blocks viral replication in productively infected cells but does not affect the reservoir of latent cells harboring proviruses that are transcriptionally silent yet capable of resuming virus production. This reservoir is responsible for viral rebound following ART cessation and is a major obstacle to curing HIV. Therapeutic approaches are being designed to target intrinsic innate immunity of latently infected cells to destroy this reservoir [2], though none have resulted in virus clearance in an HIV-infected patient.

Activation of intracellular innate immunity is critical for control of HIV infection in CD4 + T cells and myeloid cells [3, 4]. This response is initiated by pathogen recognition receptors (PRRs) that recognize and bind viral replication products known as pathogen associated molecular patterns (PAMPs). In the context of productive HIV infection, studies have shown that HIV RNA and DNA products can be recognized as PAMPs through the actions of multiple PRRs including cGAS [5], IFI16 [6], TLRs [7, 8], DDX3 [9], and RIG-I [10, 11]. Upon PAMP engagement these PRRs undergo signaling activation to direct downstream actions of transcription factors such as interferon regulatory factor (IRF)3 and NF-ΚB, which mediate expression of antiviral and immune modulatory genes, including type I and III interferons (IFNs) and inflammatory cytokines [12]. IFNs signal through their cognate receptor to induce hundreds of interferon-stimulated genes (ISGs) whose products have antiviral and immune regulatory actions to locally and systemically limit virus replication and spread [13].

Many ISGs specifically function as HIV restriction factors including *APOBEC3G* [14], *IFITM1* [15], *MX2* [16, 17], *ISG15* [18], *BST2* (tetherin) [19], and others [4, 20–22]. HIV has evolved a myriad of mechanisms to block and/or evade these restriction factors, largely through the actions of viral protein products including the HIV protease, Vif, Nef, and Vpu [4, 23, 24]. ISG restriction of viral replication and subsequent viral escape is an important area of ongoing research, though much of this work has focused on productively infected cells with ongoing viral replication. It remains unclear how actions of these ISGs might affect the transition to or reversal from latency, or what viral mechanisms of immune escape might be employed in a latently infected cell.

The type 1 IFN response significantly impacts the course of HIV infection in vivo, though this response counterintuitively can be beneficial or pathogenic, with differential results based on the timing of infection. IFNα is detectable in human plasma following HIV transmission [25], leading to an IFN response that can restrict viral replication and direct cell death to limit viral spread [4, 26–28]. IFNα from plasmacytoid DCs inhibited the establishment of latent infection in CD4 + T cells in vitro [27]. This initial IFN response imparts selection for IFN-resistant viral variants [29], however role of these viral variants in HIV latency is not known, and IFN resistance might only occur as a transient phenotype [30]. In the SIV (simian immunodeficiency virus) model, intravenous IFNα2a administered before or shortly after infection was associated with elevated ISGs including *MX2* and *OAS1*, as well as reduced virus transmission and slow disease progression in rhesus macaques [31]. However, continued treatment with IFNα2a led to reduced ISG expression and increased reservoir size in these animals, suggesting an IFN desensitized state during chronic infection. In humans, therapeutic administration of IFNα during chronic HIV infection failed to reduce disease progression [32]. Moreover, two independent studies in humanized mouse models of HIV infection demonstrated a decrease in inflammation and a reduction of the latent reservoir upon blocking of IFNα signaling [33, 34]. These studies show that IFN has dynamic effects on HIV infection and the latent reservoir in vivo, and suggest that HIV latency may be associated with reduced susceptibility to innate immune activation and/or IFN signaling.

A recent single cell sequencing transcriptomics study of T cells from HIV-infected patients showed that reactivated, latent cells express a transcriptional program that is distinct from autologous uninfected cells and includes dampened ISG expression [35], suggesting that HIV latency might impact PRR response programs. Examination of the cGAS/STING pathway in various latently infected cell lines and primary CD4 + T cells demonstrated that this pathway remains responsive during HIV infection and latency [36, 37]. Conversely, RIG-I activation by HIV RNA in latently infected cells required additional stimulation with an agonist that upregulated RIG-I signaling proteins, suggesting this pathway may be generally suppressed in latent infection [11]. Together, these studies suggest that HIV latent infection may be associated with disrupted viral RNA sensing, interferon signaling, and/or ISG activation.

Here we conducted a study to compare various in vitro models of HIV latency. We examined the response to RNA PAMP stimulation, type 1 IFN treatment, and the expression of ISGs using an integrated study design that includes established CD4 + T cell line models of HIV latency, latent infection of CD4 + T cell lines with a dual color reporter virus, and a primary CD4 + T cell model of HIV suppression. We evaluated IFN induction of a panel of antiviral ISGs in each of these models, and assessed activation of key components of innate immune and IFN signaling pathways. Using a functional genomics approach including bulk mRNA sequencing and single cell RNA sequencing, we define differences in innate immune activation and IFN signaling between uninfected cells and cells with latent or suppressed HIV infection. Our observations demonstrate the specific utility of each HIV latency model, and overall support the hypothesis that latent HIV infection involves viral selection or modification of target cells to an innate immune suppressed phenotype that supports the viral reservoir.

## Results

### Analysis of type 1 interferon response in cell line models of HIV latency

**Fig. 1:**
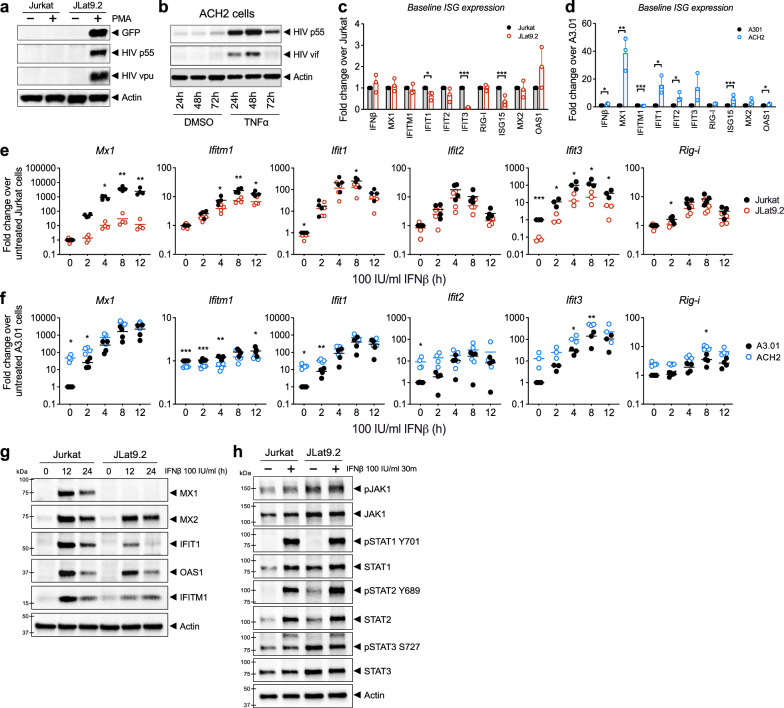
Analysis of latent cell line response to IFNβ stimulation a,b Immunoblot analysis of HIV proteins in uninfected Jurkat vs latent JLat9.2 cells treated with 16 nM PMA for 24 h (**a**), or in latent ACH2 cells mock-treated (DMSO) or reactivated with 10 ng/ml TNFα for indicated times (**b**). c,d qRT-PCR analysis of resting ISG mRNA expression in Jurkat vs JLat9.2 cells (**c**) or A3.01 vs ACH2 cells. ISG expression data for untreated cells (0 h IFN) is also shown in graphs c & c. (**d**). e,f qRT-PCR analysis of IFN-induced ISG mRNA expression in Jurkat vs JLat9.2 cells (**e**) or A3.01 vs ACH2 cells (f) treated with 100 IU/ml IFNβ for indicated times. In c-f, fold change (FC) was calculated relative to untreated, uninfected cells (ΔΔCt method), and each symbol represents mean FC of replicates from a single experiment. Data from three independent experiments are shown. Statistical significance relative to similarly treated control cells (Jurkat or A3.01) was calculated by unpaired Student’s t-test; asterisks denote significance (*p < 0.05, **p < 0.01, ***p < 0.001). g,h Immunoblot analysis of Jurkat or JLat9.2 cells stimulated with 100 IU/ml IFNβ for indicated times. Representative images from one of three independent experiments are shown. See quantification data in Additional file 1: Figure S1i, j

To examine the innate immune landscape in latent cells we utilized multiple cell line models of HIV latent infection, including two different Jurkat CD4 + T cell latency models, JLat9.2 and JLat11.1, and the CEM CD4 + T cell ACH2 latency model, each harboring silent HIV provirus [38–40]. These well-characterized latent cell lines can be compared to the uninfected, parent cells from which they were derived, and serve as useful tools for identifying pathways for further examination in physiologically relevant HIV-infection models. JLat9.2 and JLat11.1 cells were previously created by transducing Jurkat cells with a GFP-expressing lentivirus reporter, selecting for GFP induction by FACS, then culturing single cell clones to expand into a latently infected population that expresses low levels of residual HIV RNA but no detectable HIV proteins (Fig. 1a) [38]. In contrast, ACH2 cells were derived by infecting A3.01 CEM cells with HIV and then rescuing latently-infected cells; these cells constitutively express low levels of HIV RNA, proteins, and infectious virus (Fig. 1b) [40]. Unlike JLat9.2 and JLat11.1 cell lines, which each harbor a single known HIV integration, ACH2 cell populations have many unique HIV integrations [41, 42]. Both JLat and ACH2 latency models can be reactivated to induce expression of HIV proteins (Figs. 1a-b, Additional file 1: Figure S1e).

We first measured resting (baseline) mRNA levels of a variety of ISGs in these cell lines and found that most ISGs were expressed at similarly low levels in latent JLat9.2 and Jurkat cells, though several were suppressed or “downregulated” in JLat9.2 cells (*IFIT1, IFIT3, ISG15)* (Fig. 1c). Resting JLat11.1 cells similarly expressed lower *IFIT1* mRNA than Jurkat cells, though *IFITM1* mRNA was elevated at baseline (Additional file 1: Figure S1a). In contrast, ACH2 cells had elevated levels of most ISGs compared to control uninfected A3.01 cells, suggesting that persistent, low-level virus production in ACH2 cells may trigger ongoing innate immune activation (Fig. 1d).

We next assessed the IFN response in these latent cell lines compared to their respective uninfected control cell lines. Cells were treated with 100 IU/ml of IFNβ and harvested over a 12 h time course for expression analysis of a panel of ISGs. These ISGs are highly induced or “upregulated” by type 1 IFN in our control cells, have known antiviral activity against many viruses, and are known to be induced downstream of RIG-I-mediated IRF3 activation [43]. IFNβ-induced ISG mRNA and protein expression was overall considerably lower in JLat9.2 cells relative to uninfected Jurkat control cells, with *MX1, IFITM1, IFIT1, and IFIT3* induction significantly impaired (Fig. 1e, g, Additional file 1: Figure S1i). IFNβ treatment did not alter HIV expression in any latent cell line tested (Additional file 1: Figure S1c, d). *MX1* mRNA induction was also abrogated in IFNβ-treated JLat11.1 cells (Additional file 1: Figure S1b). In contrast, IFNβ-treated ACH2 and A3.01 cells both expressed high RNA levels of most ISGs, though *IFITM1* induction was limited in ACH2 cells (Fig. 1f). Thus, HIV latent infection in JLat cells is associated with aberrant regulation of IFN signaling, potentially compromising the cell’s ability to fully activate an IFN-dependent antiviral response, while ACH2 cells demonstrate elevated basal expression of innate immune genes and remain responsive to IFN.

We next evaluated expression of IFN signaling proteins and found that surface expression of the IFNα/β receptor chain IFNAR1 was similar between all latent and uninfected cell lines (Additional file 1: Figure S1f–h). When stimulated with IFNβ, latent and uninfected cell lines also increased abundance of phosphorylated STAT1 and STAT2 (Fig. 1h, Additional file 1: Figure S1j), suggesting that in this model, HIV latency does not block type 1 IFN signaling of JAK/STAT pathway activation. Interestingly, JLat9.2 cells express higher baseline levels of the phosphorylated form of STAT2 than uninfected cells (Fig. 1h, Additional file 1: Figure S1j). Taken together, these data show that HIV latent infection is associated with basal activation and aberrant regulation of IFN signaling in these cell line latency models.

### Latent HIV infection disrupts IFN responses downstream of PRR signaling

**Fig. 2:**
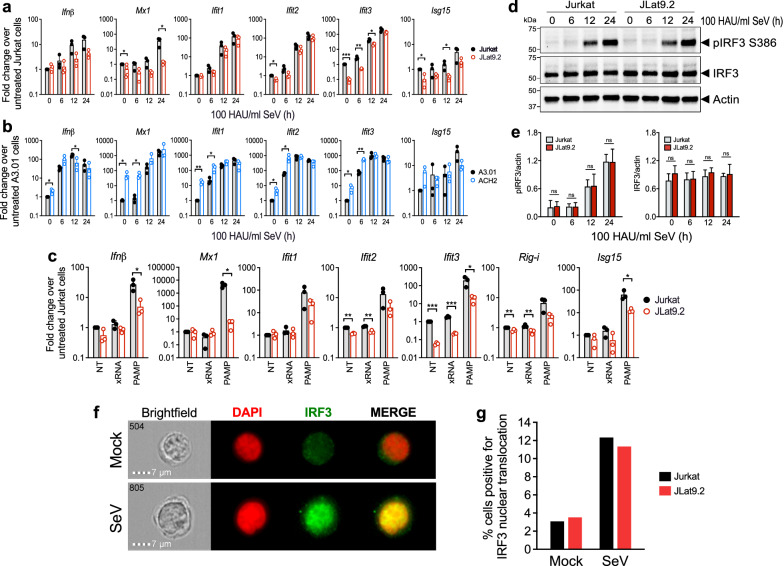
Latent HIV infection disrupts IFN responses downstream of PRR signaling a,b qRT-PCR analysis of Jurkat vs. JLat9.2 cells (**a**) or A3.01 vs. ACH2 cells (**b**) after infection with 100 HAU/ml Sendai virus (SeV) for indicated times. **c** qRT-PCR analysis of Jurkat vs. JLat9.2 cell lines left untreated (NT), or transfected with nonstimulatory xRNA or RIG-I stimulatory PAMP RNA for 24 h. Both RNAs contain 5'-triphosphate. For all qRT-PCR data (panels a-c), fold change (FC) was calculated relative to untreated, uninfected cells (ΔΔCt method), and each symbol represents mean FC of replicates from a single experiment. Data from three independent experiments are shown. Statistical significance relative to similarly treated control cells (Jurkat or A3.01) was calculated by unpaired Student’s t-test; asterisks denote significance (*p < 0.05, **p < 0.01, ***p < 0.001). **d**, **e** Immunoblot analyses of total and phosphorylated IRF3 (pIRF3) in Jurkat or JLat9.2 cells infected with 100 HAU/ml SeV for indicated times. One representative image is shown (**d**) from three independent experiments quantified (**e**). Target protein abundance relative to actin was quantified with ImageJ software, and statistical significance of JLat9.2 relative to similarly treated Jurkat cells calculated by two-way ANOVA with multiple comparisons (Holm-Sidak) (p < 0.5; ns = not significant). f,g ImageStream quantification of IRF3 nuclear localization in mock-treated or SeV-infected (100 HAU/ml, 24 h) Jurkat or JLat9.2 cells. One ImageStream experiment was performed with 20,000 cells per sample. Representative Imagestream images of brightfield, red (DAPI-stained nuclei), green (IRF3), and red/green merged images. For each cell, IRF3 nuclear translocation status (positive or negative) was determined by IRF3/DAPI similarity over an arbitrary cutoff value of 2.3 (see Imagestream gating in Additional file 1: Figure S2a)

To further evaluate viral RNA sensing and innate immune activation in latent cell lines, we infected latent JLat9.2 or ACH2 cells and control Jurkat or A3.01 cells with Sendai virus (SeV), a model RNA virus that drives robust IRF3 activation through multiple innate immune pathways [44]. SeV infection does not meaningfully reactivate HIV expression in JLat9.2 cells (Additional file 1: Figure S1e). At 12 and 24 h following SeV infection (100 HAU/ml), IFNβ expression was lower in latent JLat9.2 and ACH2 cells than in corresponding Jurkat and A3.01 controls, indicating that SeV-induced antiviral innate immune response is diminished in JLat9.2 and ACH2 latent cell lines (Fig. 2a, b). Compared to SeV-infected control Jurkat cells, SeV-infected JLat9.2 cells expressed variable but overall lower mRNA levels for all ISGs tested (Fig. 2a). SeV-infected ACH2 and A3.01 cells transcribed similar levels of most ISGs (Fig. 2b), despite the much higher baseline ISG expression in ACH2 cells (Fig. 1a). We further examined innate immune activation and ISG expression in Jurkat and JLat9.2 cells after transfection with RIG-I agonist PAMP RNA compared to nonsignaling xRNA control [45]. PAMP RNA but not xRNA induced IFNβ expression in both cell lines 24 h after treatment, and induced expression of a panel of ISGs in Jurkat cells; however, ISG expression was suppressed in JLat9.2 cells (Fig. 2c).

IRF3 activation and IFNβ expression are markers of innate immune activation that drive downstream ISG expression following PRR signaling. Productive HIV infection has been shown to drive PRR signaling but suppress IRF3 activation [46, 47]. To evaluate IRF3 activation in HIV latent cells, we assessed IRF3 phosphorylation and nuclear translocation in latent JLat9.2 and control Jurkat cells after infection by SeV. IRF3 was present in a resting state and abundant to similar levels in Jurkat and JLat9.2 cells, and was phosphorylated at serine 386 (pIRF3 S386) in both cell lines upon SeV infection (Fig. 2d, e). Imagestream analysis revealed that SeV infection triggered similar levels of IRF3 nuclear translocation in Jurkat and JLat9.2 cells (Fig. 2f, g, Additional file 1: Figure S2a). Together these results reveal that HIV latent cells are not generally impaired in PRR signaling or IRF3 activation, but instead exhibit reduced IFN production as well as suppression of IFN-induced ISG expression occurring downstream of IFN production/signaling.

### Global gene expression and analysis of the innate immune transcriptome in latent JLat9.2 cells

**Fig. 3:**
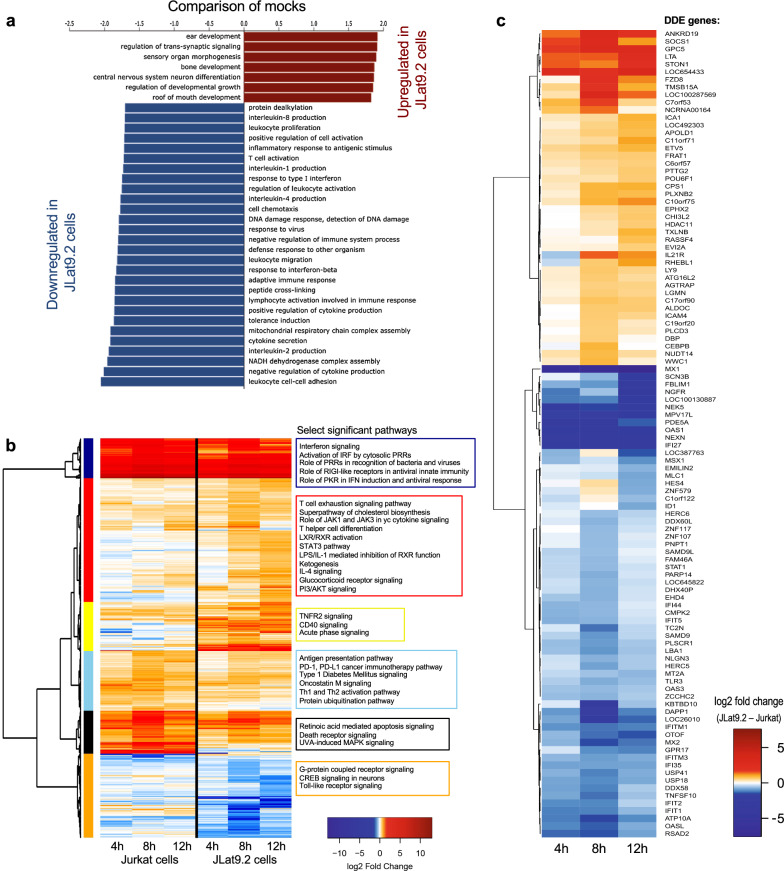
Analysis of innate immune transcriptome in latent JLat9.2 cells. **a** Gene set enrichment analysis (GSEA) of differentially expressed gene function modules between untreated (mock) Jurkat and JLat9.2 cells. Genes were ranked using a t statistic. **b** Differential expression (DE) analysis of IFN-induced genes in Jurkat or JLat9.2 cells after mock treatment (media, 4 h) or stimulation with 100 IU/ml IFNβ for 4 h, 8 h, or 12 h. One experiment was performed with three biological replicates per treatment condition. Heat map shows significant ISGs in IFNβ-treated Jurkat cells relative to mock-treated Jurkat cells (left) or IFNβ-treated JLat9.2 cells relative to mock-treated JLat9.2 cells (right). FC > 1.5, p < 0.05. Significantly enriched pathways (Ingenuity) were grouped into 6 modules depicted by colored blocks on the left, and annotated on the right with select highly significant pathways. All identified genes and pathways are listed in Additional file 2: Table S1. **c** Differential of differential expression (DDE) analysis comparing expression of IFN-induced genes in JLat9.2 cells relative to Jurkat cells treated as described above for panel b. For expression analyses in panels b and c, FC > 1.5, p < 0.05

To globally define the extent of dysregulated ISG expression in latent HIV infection we performed bulk RNA sequencing (RNAseq) transcriptomic analysis on JLat9.2 and Jurkat cells as models of HIV latency and uninfected control cells, respectively. Jurkat and JLat9.2 cells were left untreated (mock treatment) or were stimulated with 100 IU/ml IFNβ for 4, 8, or 12 h, and then analyzed by RNAseq. Three independent biological replicates were included for each experimental condition. A principal component analysis of all samples is shown in Additional file 1: Figure S3a, validating the similarity of biological replicates and revealing the distinct transcriptomic response of each cell line to IFN. We first determined the differentially expressed (DE) genes between resting (mock-treated) Jurkat and JLat9.2 cells, and grouped these genes into functional categories using Gene Ontology (GO) analyses (Fig. 3a). In Jurkat cells these DE genes clustered into GO categories that typically span ISG functions, including innate immune activation, inflammatory response, cytokine production, and immune regulation [43, 48]. In contrast, GO categories significantly enriched in JLat9.2 cells included development and differentiation programs but lacked innate immune, antiviral, or immune activation functions. These observations indicate that uninfected Jurkat cells could be more effective at activating and regulating an IFN-induced antiviral response than JLat9.2 cells which harbor HIV provirus.

We next identified genes that were induced by IFNβ (ISGs) in Jurkat or JLat9.2 cells. These ISGs were defined as genes whose expression was significantly changed (Log2 fold change > 1.2) following 4, 8, or 12 h of IFNβ treatment compared to mock-treated cells. We identified 503 DE ISGs in IFNβ-treated Jurkat and/or JLat9.2 cells within the treatment time course, shown in the heat map in Fig. 3b (see also Additional file 2: Table S1). Significantly enriched pathways were identified and grouped into six major modules, which are depicted by distinct colors in Fig. 3b, and are annotated with select highly significant pathways (see Additional file 2: Table S1 for all pathways). While IFNβ treatment induced transcriptional changes in both cell lines, the heat map shows broad differences in IFN-induced gene expression between Jurkat and JLat9.2 cells, indicating these cell lines respond differently to type 1 IFN treatment. There is an overall pattern of reduced log-fold change gene expression in JLat9.2 cells, particularly in the dark blue module (Fig. 3b; PRR activation and IFN signaling pathways), black module (retinoic acid and death receptor signaling), and orange module (GPCR and TLR signaling). Moreover, we observed a pattern of enhanced immune-regulatory and inflammatory genes in JLat9.2 cells (Fig. 3b, red and yellow modules), which include pathways related to T cell exhaustion, STAT3 signaling, and TNFR2 signaling. Taken together, these findings demonstrate the differential regulation of ISGs between JLat9.2 and Jurkat cells that link with HIV latency in this model system.

To determine how IFN response signatures diverge between these latent and uninfected cell models, we identified ISGs that were induced differentially between JLat9.2 and Jurkat cells. This "differential of differential expression" ("DDE") analysis reveals the ISG correlates of HIV latency. We identified 106 DDE genes (Fig. 3c, Additional file 2: Table S1). Remarkably, many canonical antiviral ISGs exhibited greater induction in Jurkat cells compared to JLat9.2 cells (66 genes), for example *MX1, OAS1, IFI27, STAT1, MX2, RSAD2,* and *IFITM3*, as well as additional ISGs that regulate cell proliferation/survival, such as *MT2A* [49]. The DDE heat map shows reduced expression of these 106 genes in JLat9.2 cells relative to Jurkat (see Fig. 3c)*.* Network analyses link these genes to STAT1 and STAT2 regulatory nodes (Additional file 1: Figure S3b). We also found that several innate immune regulatory genes were upregulated by IFN in JLat9.2 but not Jurkat cells, such as *SOCS1*, a known suppressor of STAT1 signaling that can block *OAS1* and *MX1* expression [20, 50]. On the other hand, IFN-treated Jurkat cells expressed higher levels of several immune activating genes that likely enhance the antiviral actions of IFN over JLat cells (Fig. 3c, Additional file 1: Figure S3b, Additional file 2: Table S1). These include *HERC5,* which encodes a major E3 ligase that supports the antiviral actions of *ISG15* [51]*,* and *PLSCR1*, a transcription co-factor of ISGs [52]. Overall, these data show that compared to Jurkat cells, JLat9.2 cells induce lower levels of many antiviral ISGs and have altered expression of IFN control genes.

### Differential ISG expression in latently infected vs. productively infected Jurkat cells

**Fig. 4:**
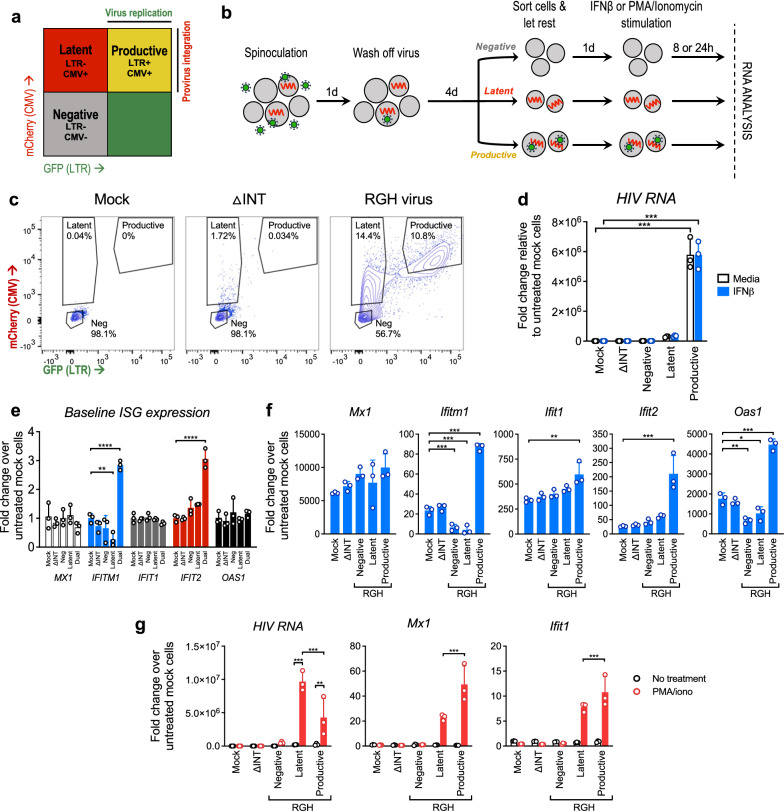
Differential ISG expression in latently vs productively infected Jurkat cells. **a** Diagram of fluorophore expression distinguishing Red-Green-HIV (RGH) virus integration vs. replication. mCherry is stably expressed by the CMV promoter, and GFP is conditionally expressed by the HIV LTR promoter upon HIV replication. **b** Schematic of the RGH virus transduction and sort protocol. Jurkat cells were mock treated, or were transduced with a ΔINT control virus or the RGH virus at MOI 0.2, then after 5d were sorted based on mCherry and GFP fluorophore expression according to the gating scheme shown in **c** (see also Additional file 1: Figure S2b). Sorted cells were rested in culture for 24 h, then cultured with media alone (untreated control), stimulated with IFNβ, or reactivated wtih PMA/ionomycin, and RNA analyzed by qRT-PCR. **d** qRT-PCR analysis of HIV RNA in transduced and sorted Jurkat cells treated with media (white bars) or 100 IU/ml IFNβ (blue bars). **e** qRT-PCR analysis of baseline ISG expression in transduced, sorted Jurkat cells. **f** qRTPCR analysis of IFN-induced ISG expression in transduced, sorted Jurkat cells treated with 100 IU/ml IFNβ for 8 h. **g** qRT-PCR analysis of transduced, sorted Jurkat cells treated with media (black symbols) or reactivated with 16 nM PMA and 1 μM ionomycin for 24 h (red symbols). For all qRT-PCR analyses, fold change (FC) was calculated relative to untreated, mock-transduced cells (ΔΔCt method), and each symbol represents FC of an individual biological replicate cultured and treated separately within single experiment (three replicates per treatment condition). Bars represent mean mean FC + SD. Statistical significance relative to mock control cells was calculated by twoway ANOVA (Holm-Sidak); asterisks denote significance (*p < 0.05, **p < 0.01, ***p < 0.001)

To further investigate ISG expression throughout different HIV infection states we utilized the Red-Green-HIV-1 (RGH) dual-color lentivector reporter, which enables distinction of latent and productively infected cells by flow cytometry [53]. Transduction with the replication-incompetent RGH virus provides a platform model of diverse populations of latently and productively infected cells with distinct provirus integration sites, overcoming many of the limitations of clonal latent cell line models. Cells transduced with RGH constitutively express mCherry via the cytomegalovirus (CMV) immediate-early promoter, indicating provirus integration, while expression of green fluorescence protein (GFP) under control of the viral long terminal repeat (LTR) element marks cells undergoing HIV transcription. Therefore, dual color fluorescence marks cells with integrated provirus undergoing active viral gene expression (Fig. 4a). Previous studies have also demonstrated that RGH-transduced cells establish stable latency as early as 4 days post transduction, enabling meaningful comparison of productive (dual fluorescent), latent, and negative cells in a relatively short time frame [53].

To assess ISG expression across transduced cells in the RGH model, Jurkat cells were treated with conditioned media (mock infection), the RGH virus (MOI 0.2), or an RGH-integrase mutant virus (ΔINT control, MOI 0.2) for 5 days. Cells were then sorted into negative, latent, or productive groups based on mCherry and GFP expression (Fig. 4b, c, Additional file 1: Figure S2b). Since this virus is replication incompetent and a low MOI was used, RGH-transduced cells are likely to have only 1 integrated provirus and be either latent or productive, but not both. Dead cells were identified by DAPI staining and excluded from sorted populations (Additional file 1: Figure S2b). Sorted cells were then placed in culture and treated with media alone (untreated control), stimulated with IFNβ, or reactivated with PMA and ionomycin, then gene expression analyzed by qRT-PCR.

At baseline (prior to IFN treatment), ISG expression was generally similar between mock, ΔINT, sorted negative, and sorted latent cells, though *IFITM1* and *IFIT2* levels were elevated in productive (dual fluorescent) cells (Fig. 4e). To assess the acute effects of IFN on ISG expression rather than long term effects on viral latency we chose a short treatment time course, and found that HIV RNA expression was overall unaffected by 8 h of IFNβ treatment (Fig. 4d). We measured fold induction of ISG mRNA in IFN-treated cell populations compared to IFN-untreated, mock control cells, and found that IFNβ induced similar levels of canonical ISGs in mock- and ΔINT-treated cells, suggesting that mere virus exposure does not alter ISG activation (Fig. 4f). Interestingly, IFN induction of select ISGs (*IFITM1* and *OAS1)* was reduced in latent cells but not mock control cells (Fig. 4f). This finding of select ISG suppression is consistent with our observations in latent HIV infection of JLat9.2 and JLat11.1 cell lines (see Fig. 1, Additional file 1: Figure S1), though the ISGs impaired vary between models. While both JLat9.2 and RGH-transduced latent cells are derived from the same Jurkat cell line, these latency models differ considerably in population diversity, integration sites, and longevity of latency such that these factors may contribute to differing phenotypes between models. Interestingly, productive (dual-fluorescent) cells demonstrated elevated ISGs both at baseline (Fig. 4e) and after IFN stimulation (Fig. 4f), indicating that HIV RNA and/or protein products stimulate innate immunity and enhance the cellular response to exogenous IFN. It is noteworthy that ACH2 latent cells, which also constitutively express viral products, have significantly elevated baseline levels of ISG mRNA (see Fig. 1d).

We next investigated if latent cells in this model could reactivate virus transcription and if this outcome would influence innate immune signaling. Cells were transduced with the RGH virus then sorted as described previously, were rested in culture overnight, then were reactivated with PMA/ionomycin for 24 h and analyzed by qRT-PCR. PMA/ionomycin treatment resulted in a robust increase of HIV RNA in latent cells but only a modest increase in productive cells (Fig. 4g). HIV reactivation led to induction of *MX1* and *IFIT1* in both these groups. Notably, however, the magnitude of induction of these ISGs was significantly reduced in latent relative to productively infected cells (Fig. 4g), despite the reactivated latent cells high expression of HIV RNA. Thus, in the RGH infection model, HIV latency associates with selective reduction of ISG induction in response to IFN treatment or virus reactivation.

### ISG expression analysis in a primary CD4 + T cell model of HIV suppression

**Fig. 5:**
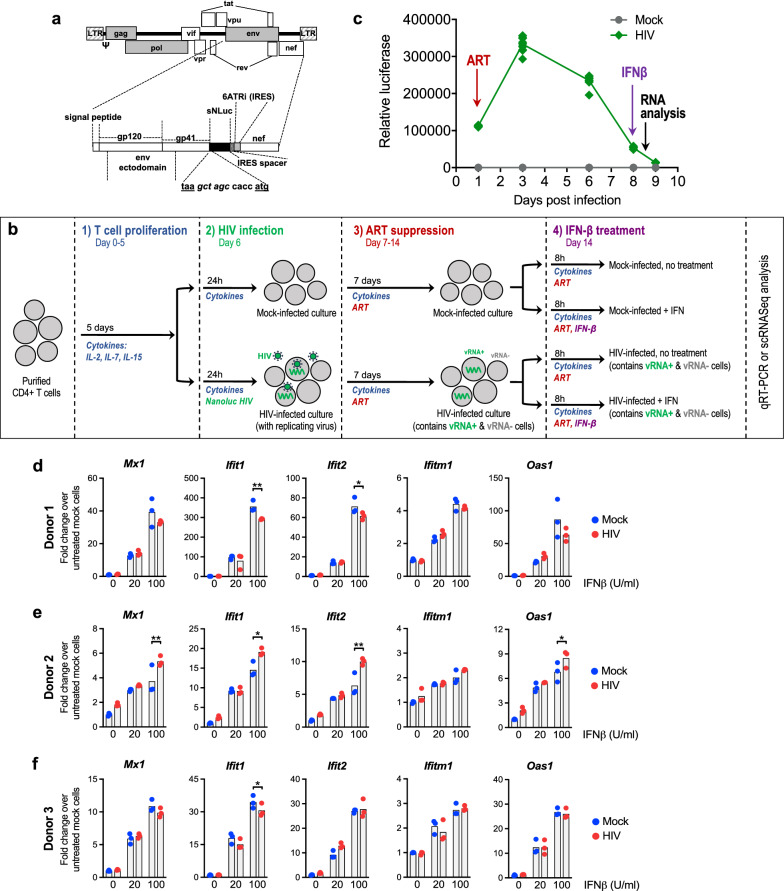
ISG expression analysis in a primary CD4 + T cell model of HIV suppression. **a** Genome organization of the NanoLuc HIV-1 reporter virus. **b** HIV infection and ART suppression protocol. Primary CD4 + T cells isolated from healthy human PBMC were cultured for 5 days in homeostatic cytokines (IL-2, IL-7, IL-15) then mock-infected (media) or infected with NanoLuc HIV at MOI 2.0 (Donors 1 & 2) or MOI 1.0 (Donor 3). 24 h after infection cells were treated with ART (10 μm raltegravir and 1 μm efavirenz) for 7 days, then stimulated with IFNβ (0, 20, or 100 IU/ml) for 8 h. **c** Luciferase expression analysis of supernatant from primary CD4 + T cells at indicated times. Three independent experiments were performed. Values represent 12 technical replicates for cells from one donor (#1) in one representative experiment (see Additional file 1: Figure S4B for additional donors). **d**–**f** qRT-PCR analyses of mock-infected or HIV-infected CD4 + T cells after IFNβ treatment. Bars represent mean FC ± SD of IFN-stimulated relative to untreated, mock-infected cells. Multiple independent experiments were performed, and data is shown from one representative experiment with three biological replicates per treatment condition. Statistical significance between mock-infected and HIV-infected cells was determined by two-tailed T test with multiple comparisons (Holm-Sidak). *p < 0.05, **p < 0.01, ***p < 0.001

We evaluated the impact of ART-mediated HIV suppression on cell-intrinsic antiviral responses in a primary CD4 + T cell model of HIV infection. We established a primary cell model of virologic suppression [54], which is similar to models from other laboratories [55–58]. This model allows analysis of HIV-infected cells in the context of low or no detectable HIV RNA expression as a proxy for HIV latency in patients undergoing ART. We treated healthy human CD4 + T cells from three different donors with homeostatic cytokines (IL-2, IL-7, and IL-15) for 5 days to increase cell permissiveness, then infected cells with a replication competent, NanoLuc reporter HIV that expresses luciferase upon HIV LTR transcription (Fig. 5a, b). At 24 h post infection, HIV infected cells transcribed HIV RNA (Additional file 1: Figure S4a) and produced luciferase indicating viral replication (Fig. 5c, Additional file 1: Figure S4b). At this time, we treated cells with ART (10 μM raltegravir and 1 μM efavirenz) to inhibit integration and reverse transcription. Luciferase production peaked at 4 days after infection then declined similar to mock levels after 7 days of ART, indicating suppression of viral replication (Fig. 5c, Additional file 1: Figure S4b). Droplet digital PCR (ddPCR) analysis showed that after 7 days of ART, approximately 3.1% of cells were positive for HIV DNA (average of 3 donors, see Additional file 1: Table S3).

To assess the IFN response in suppressed cells after 7 days of ART, mock and HIV-infected cells were resuspended in media containing ART and homeostatic cytokines, then stimulated with IFNβ (20 or 100 IU/ml, 8 h) or left untreated (ART and homeostatic cytokines alone). ISG mRNA expression was analyzed by qRT-PCR and fold mRNA induction was quantified relative to untreated cells. We found that HIV-infected cells trended to elevated baseline ISG RNA levels compared to mock, though these differences were generally not statistically significant (Fig. 5d–f, Additional file 1: Figure S4c). Compared to untreated cells (no IFN), both mock and HIV-infected cells upregulate ISGs in response to IFN. Select ISGs were suppressed in HIV-infected cells (Fig. 5d-f), though these differences were modest relative to our previous findings with cell line models of latency (see Figs. 1, 2, 3, 4). Because the rate of HIV infection is low in this model (approximately 3.1%, Additional file 1: Table S3), it makes sense that IFN responses are generally similar in magnitude between mock- and HIV-infected samples. The magnitude of ISG induction varied between donors, with T cells from donors 1 & 3 demonstrating greater ISG suppression (*IFIT1).*

We measured secreted luciferase from these cells (following ART and IFNβ treatment) to determine if IFNβ influences ART suppression of HIV replication in our model. Luciferase expression was similar in HIV-suppressed cells before and after IFNβ treatment (Additional file 1: Figure S4d), indicating that IFNβ did not alter HIV replication in this model. We assessed JAK/STAT signaling by performing a short course IFN treatment on suppressed cells from one donor and found that compared to mock, HIV-infected cells had elevated levels of phosphorylated STAT1 and STAT2 both at baseline and after 30 min of IFNβ treatment (Additional file 1: Figure S4e). However, after one hour of IFNβ treatment both uninfected and HIV-infected cells underwent the same extent of STAT1 & STAT2 phosphorylation, similar to results observed in Jurkat and JLat cells (see Fig. 1h, Additional file 1: Figure S1j).

To further validate our model of viral suppression, we removed ART from culture after 7 days and rested cells for 24 h before measuring secreted luciferase. Removal of ART resulted in elevated luciferase in HIV-infected cells, indicating virus rebound and validating that ART actively suppresses HIV replication in this model (Additional file 1: Figure S4f). Reactivation with PMA and ionomycin at this time point further increased luciferase expression indicating the presence of cells with reactivatable proviruses (Additional file 1: Figure S4f). Thus, viral suppression and reactivation of HIV is faithfully captured in our primary cell model, wherein ART-suppressed HIV infection associates with altered response to IFNβ for select genes, similar to our observations in cell line models of HIV latency (see Figs. 1, 2, 3, 4).

### scRNA-seq identification of HIV-regulated genes in primary CD4 + T cells

To define the relationship between a suppressed HIV provirus and innate immune defenses within primary cells on a single cell level, we examined our model of HIV suppressed primary CD4 + T cells, noting that while these cells have suppressed viral replication, they typically retain low levels of detectable HIV RNA. These properties of our culture model enable identification of infected cells through single cell RNA sequencing (scRNA-seq). CD4 + T cells from two healthy human PBMC donors were cultured in homeostatic cytokines (IL-2, IL-7, and IL-15) for 5 days, then mock-treated or infected with NanoLuc HIV (MOI 2.0, 24 h), suppressed for 7 days with efavirenz and raltegravir, then were left untreated or were treated with IFNβ (100 IU/ml) for 8 h. As in our previous experiments, we chose a short course of IFNβ treatment, which neither affects HIV transcription nor replication in this model (see Additional file 1: Figure S4d). The cultures were then subjected to scRNA-seq analysis (approximately 8,000 cells per sample). Cells identified to contain at least one read mapping to the HIV-1 genome (one UMI count) were classified as viral RNA positive (vRNA +). While we analyzed 40,000 reads per cell, this extent of mRNA reads might not be fully representative of the total cellular content of HIV mRNA. However, this method does reasonably enable distinction of vRNA + and vRNA- cells. HIV-infected samples contained 3.9–9.8% of vRNA + cells (Additional file 1: Table S3). Analysis of CD4 + T cell subsets showed that HIV RNA expression was enriched in inducible regulatory T cells (Additional file 1: Figure S5a–c).

**Fig. 6:**
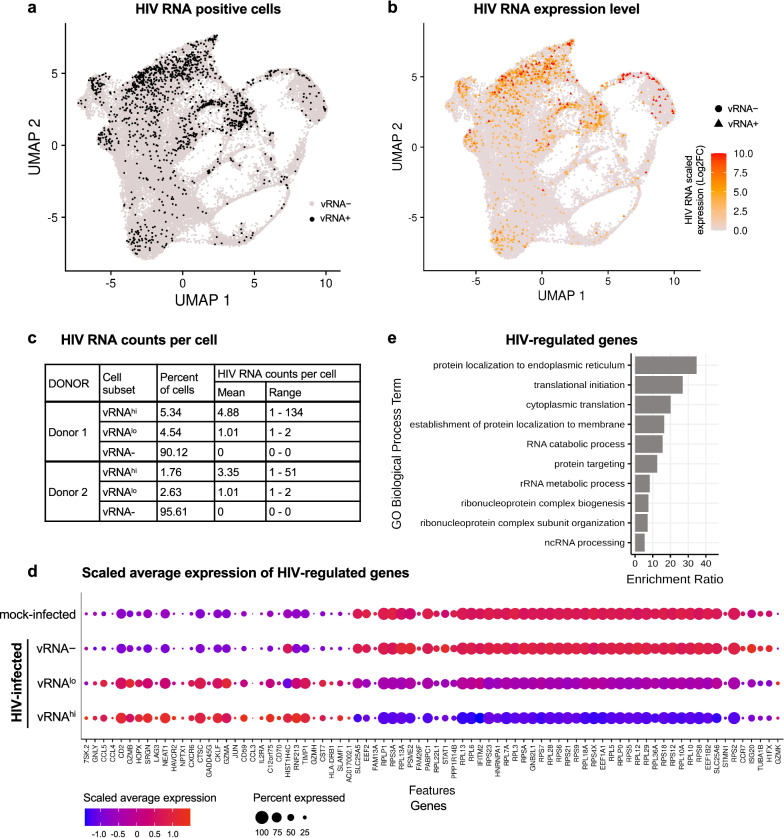
Single cell RNA sequencing identification of HIV-regulated genes in primary CD4 + T cells CD4 + T cells from two healthy human donors (#1 & 2) were mock-infected (media) or were HIV-infected (NanoLuc HIV, MOI 2.0) for 24 h, then suppressed with ART (10 μm raltegravir and 1 μm efavirenz) for 7 days and analyzed by scRNA-seq (see protocol schematic in Fig. 5b). **a**, **b** UMAPs show clustering of vRNA + cells and vRNA- cells, or viral RNA expression level per cell in all combined samples (CD4 + T cells from two human donors, mock- or HIV-infected, ± IFNβ). **c** HIV RNA counts in vRNAhi, vRNAlo, and vRNA- cell subsets for each donor. **d**, **e** HIV regulated genes identified through differential expression analysis of vRNAhi and vRNAlo cells relative to mock-infected cells (FC > 1.2, p < 0.05). 80 significant genes were identified across both donors (see Additional file 3: Table S2). Dot plot shows scaled average expression of these genes, and bar graph shows the top ten significantly enriched modules of HIV-regulated genes

vRNA + and vRNA- cells from the entire scRNA-seq data set were displayed in a uniform manifold approximation and projection (UMAP) reduction (Fig. 6a). vRNA + cells tend to cluster near each other in the UMAP suggesting a similar transcriptome profile in these cells. A second UMAP plot demonstrates differential HIV RNA expression level within vRNA + cells and reveals that these cells tend to cluster near others with similar HIV RNA abundance (Fig. 6b). Given the wide range of HIV RNA expression and the sensitivity of our scRNA-seq method to detect a single viral transcript, we further classified vRNA + cells into high- and low-viral RNA expressing subsets by first calculating the median value of HIV counts in cells with HIV, and subsequently classifying cells as vRNA^hi^ if HIV counts are above the median value and cells as vRNA^lo^ if counts are below the median value (Fig. 6c). vRNA^hi^ cells from each donor express HIV RNA levels (mean 4.9 and 3.4 HIV RNA counts) that might be expected from cells undergoing low levels of viral replication, whereas the reduced HIV RNA abundance in vRNA^lo^ cells (mean 1 HIV RNA count) suggests this population has undergone some degree of transcriptional suppression as in latent cells. While we cannot exclude the possibility of latent cells within the vRNA- subset, our ddPCR data revealed only a low total percentage HIV DNA + cells, suggesting that cells with true latent HIV infection are a minority fraction of the larger vRNA- population.

To determine how HIV influences global gene expression in the context of ART suppression, we performed DE analyses to define HIV regulated genes. We analyzed gene expression in vRNA^hi^ and vRNA^lo^ cells from untreated (no IFN) HIV-infected samples for both donors, and identified 80 HIV-regulated genes that were differentially expressed in one or both cell subsets relative to mock-infected cells (Fig. 6d, Additional file 3: Table S2). Many of these genes are involved in processes relating to RNA translation, protein localization, and protein targeting (Fig. 6e), indicating that in our primary cell model of virologic suppression, HIV infection modulates protein and RNA metabolism. Genes that were significantly elevated in both vRNA^hi^ and vRNA^lo^ subsets but not in vRNA- cells at baseline relative to mock-infected cells include *LAG3/CD223*, an inhibitory receptor of lymphocyte activation known to be induced in response to HIV infection and to contribute to HIV persistence [59–61], several granzyme-encoding genes (*GZMA, GZMB, GZMH), LGALS3* (encoding Galectin-3) which is known to be induced by HIV [62], and *RNF213*, an E3 ubiquitin ligase [63]. We found that a subset of ISGs were suppressed at baseline in HIV^lo^ or HIV^hi^ cells compared to mock-infected cells, including *STAT1, RPL5, IFITM2, IFITM3, GBP1, IFIT3, and ISG20* (described below).

### scRNA-seq analysis of ISG induction in HIV-suppressed, primary CD4 + T cells

**Fig. 7:**
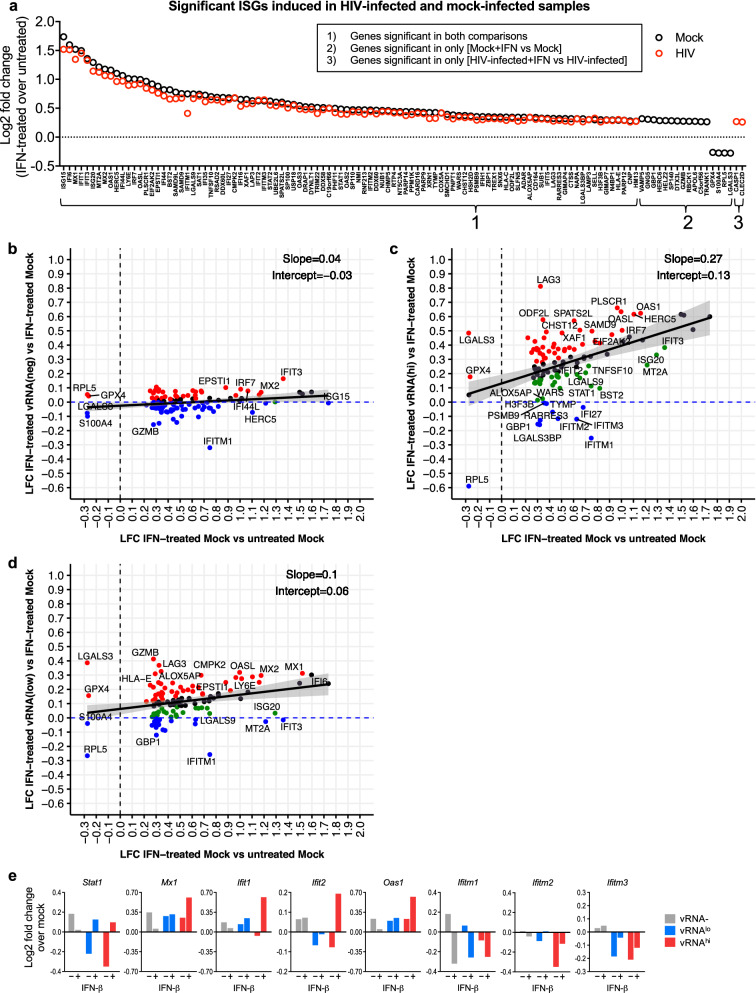
Single cell RNA sequencing analysis of ISG induction in HIV-suppressed, primary CD4 + T cells CD4 + T cells (donors #1 & 2) were mock-infected (media) or HIV-infected (NanoLuc HIV MOI 2.0) for 24 h, then suppressed with ART for 7 days. At day 8 cells were left untreated or were stimulated with 100 IU/ml IFNβ for 8 h, then analyzed by scRNA-seq (see schematic in Fig. 5b). **a** Differential expression (DE) analysis of bulk populations of IFN-treated mock vs. untreated mock cultures (black circles), and IFN-treated HIV-infected vs. untreated HIV-infected cultures (red circles). We identified 116 ISGs that were significantly differentially expressed in any IFN-treated sample relative to corresponding untreated control (Log2 fold change > 1.2, p < 0.05). **b**–**d** Log2 fold change expression of 116 ISGs in each vRNA subset from IFN-treated, HIV-infected populations (vRNAhi, vRNAlo, or vRNA-) relative to IFN-treated, mock-infected cells (see Additional file 3: Table S2). For all graphs above, dotted lines represent control to which graphed samples are normalized (**a** IFN-untreated cells; **b**–**d**, y axis: untreated mock-infected cells; **b**–**d**, x axis: IFN-treated mock-infected cells). **e** Log2 fold change gene expression in HIV-infected relative to similarly treated mock-infected cells, for select ISGs from graphs 7b–d

Since bulk RNA-seq of latent cell lines revealed broad disruption of the interferon transcriptome (see Fig. 3), we next focused our primary cell scRNA-seq analysis on IFN-responsive genes. We first performed a differential expression (DE) analysis comparing bulk IFN-treated cultures (mock-infected or HIV-infected) to each corresponding culture in the absence of IFNβ treatment (untreated). We identified 116 ISGs that were significantly induced by IFNβ in any sample relative to its corresponding untreated control, independent of vRNA status (Fig. 7a). Of these 116 ISGs, 98 were significantly induced by IFN in both mock-infected and HIV-infected cultures, 16 were significant only in mock-infected cultures, and 2 were significant only in HIV-infected cultures. The magnitude of ISG induction exhibited the general trend of being slightly reduced overall in cells from HIV-infected cultures, which is similar to our qRT-PCR analysis of this model (see Fig. 5d–f). These ISGs were identified through combined analysis of both T cell donors. The average expression of ISGs for individual donor samples is shown in Additional file 1: Figure S6. Since a small percentage of cells actually harbors HIV RNA in the HIV-infected cultures (see Additional file 1: Table S3), it follows that this bulk population of cells would generally mimic the mock-infected population, underscoring the value of single cell approaches.

We next applied our single-cell sequencing approach to assess how the cellular response to IFN is influenced by specific HIV infection status. We measured ISG expression at the single-cell level in three previously identified subpopulations from HIV-infected cultures (vRNA^hi^, vRNA^lo^, or vRNA- cells; see Fig. 6a–c) after 8 h of IFNβ treatment. ISG expression in the vRNA^hi^, vRNA^lo^, or vRNA- cells are then separately compared to gene expression in IFN-treated mock cells (Fig. 7b-d, respectively). Each of these three graphs present expression dynamics of the same set of 116 ISGs from Fig. 7a. The x-axis of each graph depicts Log2 fold change (LFC) ISG expression between IFN-treated mock cells vs untreated mock cells, equivalent to black symbols in Fig. 7a. The y-axis depicts the LFC of ISG expression between IFN-treated cells from HIV-infected cultures (vRNA^hi^, vRNA^lo^, or vRNA- cells) vs IFN-treated mock-infected cells. For each condition (IFN treated HIV vRNA^hi^, vRNA^lo^, or vRNA- vs IFN mock) we performed linear regression modeling of the LFCs to LFCs from the IFN-treated vs mock thereby defining the impact of HIV on ISG expression and how this relates to ISG expression in IFN-treated mock-infected control cells. For each linear regression model, we generated a 95% confidence interval (CI; shown as the grey-shaded region on each graph). The CI depicts with 95% confidence where ISG LFCs across the set of 116 ISGs would be expected to fall given high, low or negative levels of vRNA (y axis) be present in the presence of IFN given their response to IFN (x axis). As noted in the previous analysis (see Fig. 6), here we similarly combined data from both CD4 + T cell donors.

We identified differential and variable ISG expression across all three HIV vRNA status conditions in our primary cell model. IFN-treated vRNA- cells demonstrated a pattern of ISG expression that was generally similar to IFN-treated mock-infected cells (Fig. 7b). The regression model had a slope of 0.04 suggesting that HIV exposure without detectable infection does not meaningfully alter ISG regulation after 8 h of IFN treatment in this model system. However, 24 ISGs (21% of ISGs) fell below the y-axis zero line (Fig. 7b, blue symbols), indicating their reduced expression in the IFN-treated vRNA- cells relative to IFN-treated mock-infected cells. The overall trend of ISG response to IFN was quite different in vRNA + cells (Fig. 7c, d). The slopes of the linear models in these graphs have stronger positive values (0.27 and 0.1 for vRNA^hi^ and vRNA^lo^, respectively) and shifted positively on the y- axis (intercept value of 0.13 and 0.06 for vRNA^hi^ and vRNA^lo^ respectively), suggesting most of these genes have enhanced induction in response to HIV in response to IFN. Especially interesting are ISGs that fell outside the CI and below the y-axis 0-line, indicating their downregulation in IFN-treated vRNA^hi^ or vRNA^lo^ cells relative to IFN-treated mock-infected cells (Fig. 7c, d; Additional file 3: Table S2).

A deeper analysis of the data presented in Fig. 7c, d reveals distinct differences in IFN response between vRNA^hi^ and vRNA^lo^ cells. ISG mRNA expression was overall considerably augmented in IFN-treated vRNA^hi^ cells relative to all other cells, suggesting that higher levels of viral products might enhance IFN responses, as demonstrated in both ACH2 cells (see Fig. 1) and productive cells from the RGH model (see Fig. 4). In contrast, IFN-treated vRNA^lo^ cells had variable levels of ISG mRNA expression compared to similarly treated mock-infected cells (Fig. 7d). This outcome contrasts with our findings in cell line latency models which demonstrate significantly impaired ISG induction after IFN treatment. However, we identified a subset of ISGs poorly induced by IFN in HIV-infected (vRNA^hi^ and/or vRNA^lo^) cells, which may have altered regulation in the context of HIV infection. These are depicted by blue symbols in Fig. 7c, d and include *IFITM1, IFITM2, IFITM3, GBP1, MT2A,* and *LGALS3BP.* Resting and IFN-stimulated expression of select ISGs from these figures are also shown in Fig. 7e. Overall, the single cell transcriptome of primary CD4 + T cells under ART conditions of HIV suppression reveals that vRNA^hi^ and vRNA^lo^ cells display altered baseline expression and IFN-induced expression of select ISGs.

We integrated our transcriptomics data sets to identify HIV-regulated genes across our various cell culture infection models, summarized in Additional file 1: Table S4. These are ISGs that were identified as significantly differentially expressed between latent or HIV-suppressed cells and corresponding controls across our cell models. This gene set includes *GBP1, IFIT1, IFIT2, IFITM1, IFITM2, IFITM3, ISG15, MT2A, MX1, OAS1, RPL5, RSAD2 (viperin),* and *STAT1*. Because these genes demonstrated significantly different expression and/or IFN induction between uninfected and latent cells in our culture models, we propose these genes are worthy of further study to reveal possible novel functions in which their expression could otherwise mitigate the establishment or persistence of latent HIV infection.

## Discussion

In this study we examined innate immune activation and IFN response in cell line and primary cell models of HIV latency. Innate immunity plays an important role in early control of HIV infection in vivo, but it is not well understood if latently-infected cells have functional innate immune and interferon responses. Here we investigated responses to RIG-I agonists and type 1 interferon in a variety of different in vitro models of HIV latent infection or virologic suppression, including a model of HIV suppression in primary CD4 + T cells. When stimulated with RIG-I agonists or IFN, J-Lat and ACH2 latent cell lines demonstrate reduced ISG induction compared to uninfected control cells, suggesting that the establishment of long-term latency in these models may be linked with a dysregulated response to IFN downstream from IRF3 activation. A similar outcome was observed in Jurkat T cells that were latently infected with a dual color reporter HIV. Primary CD4 + T cells infected with HIV and suppressed by ART demonstrate variable but reduced responses to IFN stimulation compared to mock-infected cells across a panel of 116 ISGs. Single cell transcriptomic profiling of these HIV-suppressed CD4 + T cells revealed that viral RNA positive (vRNA +) and negative (vRNA-) cells from the same culture have distinct gene expression profiles, with altered expression of genes involved in RNA processing, translation, and IFN response. Further analysis of vRNA + cells revealed that the magnitude of ISG induction after IFN stimulation was lower in cells expressing low HIV RNA (vRNA^lo^) than cells expressing higher viral RNA (vRNA^hi^). Our observations in multiple models of HIV latency or primary cells undergoing virologic suppression suggest that HIV latent infection is associated with transcriptional changes that alter type 1 IFN responses, though the specific genes altered vary between models.

The HIV latent reservoir in vivo is a diverse population that includes multiple CD4 + T cell subsets, distributed in different tissues, and with distinct proviral integration sites. A major challenge to understanding HIV latency has been the availability of in vitro models that capture the heterogeneity of the HIV reservoir. We compared a variety of HIV latency models representing various aspects of latent infection or viral control, and investigated the relationship of IFN response and HIV infection across these models. Latent cell lines are clonal populations that proliferate, representing latent cells that might arise from clonal expansion in vivo, but each cell line only represents a single unique latent cell with a single proviral integration. In contrast, the RGH fluorophore reporter system models acute infection with heterogenous provirus integration, and enables comparison of latent and productive cells, though virus-driven transcriptional repression may impact fluorophore expression and influence the integrity of each population. On the other hand, our primary cell model represents productive infection of activated CD4 + T cells that are then suppressed by ART. While this model approximates HIV latency it does not represent latent infection arising from complete transcriptional silencing or clonal expansion. However, this model represents a diverse population of primary cells and accounts for the effects of ART, mimicking the diverse reservoir of ART-treated patients in a context of HIV suppression and latency. We reveal that latent infection links with altered type 1 IFN responses, though phenotypes varied between the different latency models tested. We also identified distinct transcriptional changes within the IFN-induced ISG network in each model. Considering the vast biological diversity of the latently infected cell population in vivo [64], it is likely that IFN response regulation might also occur through multiple mechanisms. These mechanisms are not limited to virus-mediated processes, but might also be linked with intrinsic cell-specific differences in IFN signaling and response programs across CD4 + T cells that serve the basis for supporting latent HIV infection in a manner selected by HIV.

Our analysis of innate immune activation and IFN signaling in latent cell lines showed that latent cells stimulated with RIG-I agonists or IFN induce lower levels of select ISGs than uninfected cells, notably *MX1*. However, the ISGs altered varied between latency models. Since Sendai virus induced similar levels of IRF3 phosphorylation and nuclear translocation in latent JLat9.2 and control Jurkat cells, we conclude that RIG-I signaling and IRF3 are functional in this model. Furthermore, we observed no loss of IFNAR1 expression or JAK/STAT phosphorylation in latent cell lines, suggesting that processes that suppress ISG expression might occur downstream of these factors, possibly at a transcriptional level. Indeed, latent HIV infection drives many cellular changes that could contribute to transcriptional repression of select ISGs, including modulation of transcription factors like STAT1, NF-ΚB, and IRF1 [65], suppressive chromatin modifications that downregulate proviral genes [66], and alteration to ribosomal proteins [67].

Our functional genomics analyses showed that latent cell lines and HIV suppressed primary cells had reduced mRNA abundance for select ISGs compared to uninfected cells such that numerous ISGs were expressed to lower baseline levels and poorly induced by IFN in these latency models, as summarized in Table 1. Our single cell sequencing analyses also revealed that HIV-suppressed cells with detectable HIV RNA (vRNA +) had altered levels of many genes involved in RNA biogenesis, RNA metabolism, and translation processes, compared to mock-infected cultures or viral RNA negative (vRNA-) cells from the same population. These observations support a model in which HIV infection and latency drives changes to RNA transcription and translation machinery, potentially impacting the expression of ISGs.

**Table 1:** Candidate IFN-responsive latency restriction factors

Gene	scRNA-seq (Fig. 7B–D)	Bulk RNA-seq DDE (Fig. 3B, C)	qRT-PCR studies (Figs.1 , 2)	Gene function	References
GBP1	Suppressed in vRNA^hi^ & vRNA^lo^ cells*	Not significant	Not tested	Inhibits viral glycoproteins	[99],[100]
**IFIT1**	Not significant	Suppressed in JLat9.2*	Suppressed in JLat9.2*	Inhibits viral RNA expression	[68]
**IFIT2**	Not significant	Suppressed in JLat9.2*	suppressed in JLat9.2*	Inhibits viral RNA expression	[68]
**IFITM1**	Suppressed in vRNA^hi^ & vRNA^lo^ cells*	Suppressed in JLat9.2*	Suppressed in all latent cell lines* and RGH latent cells*	Blocks viral fusion and release	[15] [101] [20]
IFITM2	Suppressed in vRNA^hi^ & vRNA^lo^ cells*	Not significant	Not tested	Inhibits viral entry & induces apoptosis	[15] [101] [20]
**IFITM3**	Suppressed in vRNA^hi^ & vRNA^lo^ cells*	Suppressed in JLat9.2*	Not tested	Inhibits viral entry	[15] [101] [20]
ISG15	Not significant	Suppressed in JLat9.2*	Suppressed in JLat9.2* and ACH2 cells	Isgylates antiviral proteins to enhance/inhibit activity; antiviral activity against many viruses	[18] [20] [51]
**MT2A**	Suppressed in vRNA^hi^ & vRNA^lo^ cells*	Suppressed in JLat9.2*	Not tested	Anti-oxidant; regulates intracellular heavy metals; regulates apoptosis	[49]
MX1	Not significant	Suppressed in JLat9.2*	Suppressed in JLat9.2* and JLat11.1*	binding/inactivation of viral ribonucleocapsid	[20]
OAS1	Not significant	Suppressed in JLat9.2*	Suppressed in all latent cell lines* and RGH latent cells*	Activates Rnase L to degrade viral RNA	[20]
RPL5	Suppressed in vRNA^hi^ & vRNA^lo^ cells*	Not significant	Not tested	Ribosome component; binds 5S RNA	[69]
RSAD2 (viperin)	Not significant	Suppressed in JLat9.2*	Not tested	Inhibits viral RNA replication; binds viral proteins; dysregulates lipid metabolism	[21]
**STAT1**	Downregulated in resting vRNA^hi^ & vRNA^lo^ cells*	Suppressed in JLat9.2*	Not tested	Critical IFN signaling effector in JAK/STAT pathway; inhibits NF-κB; potentiates TNF⍺-mediated apoptosis	[20][102]

Bold = key genes suppressed in multiple studies

*Statistically significant (p < 0.05)

A key unanswered question in HIV infection is why some productively infected cells die while others progress to latency. Compromised IFN signaling in a given cell may support latency persistence, though it is unclear if this phenotype is driven by HIV infection or is an inherent property of a pre-latent cell. The magnitude and efficacy of the intracellular innate immune response depends on many factors such as expression and abundance of signaling proteins (PRRs, STATs, IRFs, IFNAR, etc.), post-translational modifications of these proteins, and transcriptional regulation of ISGs [68]. These factors vary between individual cells and may predispose cells that have poor antiviral responses to latent infection. Indeed, our single cell sequencing study revealed that vRNA + cells (including vRNA^hi^ and/or vRNA^lo^ subsets) have a distinct transcriptional program that suppresses markers of cell proliferation (*JUN, LAMP3, RPL5*) [69–71], regulates NF-ΚB signaling (*LGALS3, RNF213*) [62, 63], and promotes expression of markers of T cell exhaustion (*LAG3*) [59] (see Additional file 3: Table S2). Some of these properties might be explained by preferential infection of Tregs over other CD4 + T cell subsets, which is shown in our model and others, and suggests that the epigenetic programming of T cells destined for resting and suppressive fates might impact latency outcomes. A recent single cell sequencing study similarly linked HIV latency in primary CD4 + T cells to T cell subset identity, finding that HIV provirus suppression was associated with a central memory transcriptional phenotype [72]. Thus, cells with immune suppressive transcriptomes, whether from stochastic expression of antiviral genes or predetermined T cell fates, may be selected for HIV latency.

Integration of data sets of our sequencing studies identified ISGs that were suppressed in latent or HIV-suppressed cells but do not yet have known roles in HIV latency (see Table 1). These include genes that were downregulated in vRNA^hi^ and/or vRNA^lo^ relative to mock-infected CD4 + T cells (*STAT1, IFITM1, IFITM2, IFITM3, GBP1, RPL5, ISG20*), or were poorly induced by IFN in one or more cell line models of latency (*STAT1, MX1, IFITM1, IFITM3, IFIT1, IFIT2, MT2A, OAS1, ISG15, RSAD2)*. *STAT1* was downregulated in latent or HIV-suppressed cells across all latency models we evaluated. Expression of this critical gene required for IFN signaling therefore negatively impacts the ability of HIV to establish latency. Remarkably, *IFITM1* was poorly induced by IFNβ in JLat9.2 cells, RGH latently infected Jurkat cells, and HIV-suppressed CD4 + T cells positive for vRNA. Single cell sequencing analysis showed that the related *IFITM2* and *IFITM3* genes were also suppressed in IFN-treated vRNA + cells, highlighting a potential role for the IFITM family proteins in HIV-1 latency restriction. We propose that the genes listed in Table 1 should be considered for study as candidate latency restriction factors. Modulation of the expression of these genes directly or indirectly by HIV could serve to promote a cellular environment that is conducive to the establishment and long-term maintenance of latency. By this model, the high or sustained expression of these specific ISGs would be predicted to disrupt one or more essential biological processes within CD4 + T cells that otherwise facilitate the establishment and/or persistence of HIV latency.

## Conclusions

We evaluated innate immune activation and IFN responses in multiple distinct in vitro models of HIV latency, and observed that latent cells have impaired induction of select ISGs. While these ISGs varied between latency models, a core set of these genes exhibited conserved regulation across RNA sequencing studies (Table 1), revealing suppression of specific ISG expression linked with HIV latency or ART-suppressed HIV models. This study highlights a role for type 1 IFN in the formation, composition, and long-term maintenance of the HIV reservoir. Here we also identified ISGs that may have unique and distinct roles in latently infected cells, though dysregulated ISGs also varied between latency models. Additional studies are needed to elucidate the functions of these ISGs and determine how they might regulate HIV latency as possible “latency restriction factors”. Our observations contribute to the understanding of how cells might be selected by HIV for latency through altered ISG expression. Our observations support the notion that restoring and/or enhancing specific ISG function in latent cells could be critical for the success of any latency reversal therapies targeting innate immune programs.

## Methods

### Cell lines

Jurkat E6-1, HEK-293 T, and TZM-bl cells were obtained from the ATCC. JLat9.2, ACH2, and A3.01 cells were obtained from the NIH Aids Reagent Program. JLat11.1 cells were a kind gift from Dr. Florian Hladik at the University of Washington. Jurkat, JLat9.2, JLat11.1, A3.01, and ACH2 cells were cultured in RPMI supplemented with 10% FBS, 1% L-glutamine, 1% non-essential amino acids, 1% sodium pyruvate, and 1% antibiotic/antimycotic cocktail (all Fisher Scientific), hereafter referred to as complete RPMI. HEK-293 T and TZM-bl cells were cultured in DMEM supplemented with 10% FBS, 1% L-glutamine, 1% non-essential amino acids, 1% sodium pyruvate, and 1% antibiotic/antimycotic cocktail. All cells were maintained at 37 °C and 5% CO2. Cell lines were thawed from early passages and kept in culture no longer than 4 weeks. All cell lines have tested negative for mycoplasma contamination.

### Primary CD4 + T cell isolation and culture

PBMC from three healthy human donors were isolated from half leuko packs (Bloodworks Northwest) by Ficoll-Paque gradient centrifugation. Briefly, cells were collected from half leuko packs and diluted in 200 ml complete RPMI with 2 mM EDTA. 30 ml of diluted PBMC were layered over 15 ml Ficoll-Paque PLUS (GE Healthcare) in 50 ml conical tubes, then centrifuged at 400xG for 30 min at 20 °C. PBMC were collected and transferred to 50 ml conical tubes, washed with complete RPMI, then residual red blood cells lysed for 5 min with RBC lysis buffer. PBMC were washed with complete RPMI and passed through mesh filters. CD4 + T cells were purified from PBMC by negative magnetic separation with a CD4 + T cell isolation kit (Miltenyi) using a QuadroMACS separator (Miltenyi) according to the manufacturer's instructions. Purified CD4 + T cells were cultured at 37 °C and 5% CO2 in RPMI media supplemented with 10% FBS, 1% L-glutamine, 1% non-essential amino acids, 1% sodium pyruvate, and 1% antibiotic/antimycotic cocktail (complete RPMI). Cells were cryopreserved in FBS containing 10% DMSO and stored in liquid nitrogen. CD4 + T cells were genotyped for the CCR5Δ32 polymorphism and confirmed CCR5 wild-type homozygous (Donor #1 & 2), or CCR5Δ32 heterozygous (Donor #3).

### Generation of replication competent NanoLuc-expressing HIV-1 reporter virus

We generated the HIV-1 infectious molecular clone (IMC) vNL-sNLuc.6ATRi-B-Bal.Ecto, a secreted nanoluciferase (sNLuc) reporter virus (hereafter referred to as "NanoLuc HIV"), which expresses the Env ectodomain of HIV-1_BaL_ within the NL4-3-derived proviral backbone, based on our previously described HIV-1 proviral constructs encoding either the sNLuc.T2A or LucR.6ATRi reporter cassettes [73, 74]. The T2A “ribosomal skip peptide” was replaced with the modified encephalomyocarditis virus (EMCV) 6ATR internal ribosome entry site (IRES) element (6ATRi), which enables physiological Nef expression and function [74–77]. We replaced the *LucR* reporter with secreted NanoLuc® [73], inserting *sNLuc* ORF upstream of 6ATRi. Upon replication, the sNLuc reporter is secreted into the culture supernatant, facilitating kinetic monitoring of infection [73]. The reporter IMC is replication competent and encodes all the viral open reading frames, allowing for multiple rounds of viral replication.

*Description of plasmid.* The ectodomain of Env BaL (Genbank accession number: AY426110.1) derives from the HIV-1 isolate BaL. In the previously described reporter IMC, pNL-LucR.6ATRi-B.BaL.ecto [74], the *Renilla luciferase* gene (*LucR*) was replaced by InFusion® (Takara Bio) cloning methods with the soluble nanoluciferase-expressing *sNLuc* gene. Fusion of the *NLuc* gene to an N-terminal secretion signal generates a secreted, 19.1 kDa, form of the NanoLuc® luciferase, secNLuc (Promega, under limited use label license). In the current IMC, the *sNLuc* IRES cassette was inserted between the NL4-3 *env* and *nef* genes. The *sNLuc* ORF is located downstream of the stop codon (taa) of *env* and a Kozak sequence (ccacc); it is followed by a 26 nt “spacer”, the IRES element and the *nef* gene. The IRES we used is derived from encephalomyocarditis virus (EMCV) (GenBank: EMCV IRES, NC_001479), contains the “wild type” (A)_6_ (“6A”) bifurcation loop, and encompasses a truncated EMCV IRES fragment (“TR”, nucleotides 399 to 833). The proviral plasmid was generated and provided by Dr. Christina Ochsenbauer (University of Alabama at Birmingham, Department of Medicine).

### Preparation of virus stocks

Sendai virus (SeV) Cantrell Strain stock was obtained from Charles River Laboratories.

The Red-Green-HIV (RGH) virus and ΔINT control virus were generated from the following viral molecular clones obtained from the NIH AIDS Reagent Program: pRGH-WT and pRGH-Integrase D116A (ΔINT). The RGH virus stably expresses mCherry under the control of a CMV promoter, and conditionally expresses GFP under the control of the HIV LTR promoter upon productive HIV replication [53]. Vesicular stomatitis virus G (VSV-G) pseudotyped stocks were generated by transfecting HEK293T cells with viral molecular clones and pHEF-VSVg in a 10:1 ratio using the Fugene HD Transfection Reagent according to manufacturer's instructions. Cell supernatant containing virus was collected at 48 h post infection and virus titer determined on TZM-bl cells.

The NanoLuc HIV viral stock was generated by Dr. Rena Astronomo and collaborators at the Vaccine Infectious Disease Division, Fred Hutchinson Cancer Research Center (Seattle, WA). The methods and reagents used for the generation of virus stock and calculation of virus infectivity were described previously [73]. In brief, the vNL-sNLuc.6ATRi-B.Bal.Ecto reporter virus was generated by transfection of proviral DNA into 293 T/17 cells (ATCC) using Lipofectamine 2000 according to the manufacturer’s protocol (Thermo-Fisher). Viral supernatants were harvested 60 h post-transfection, clarified at 1200 × g for 10 min, and frozen at − 70 °C. Virus stocks were analyzed for nanoluciferase expression using Nano-glo luciferase (Promega) and were titered on sub-confluent TZM-bl cells (NIH ARP). Virus was diluted in DMEM supplemented with 1% FBS and 40 µg/ml DEAE-Dextran and added to cells for 4 h. Growth medium (DMEM, 10% FBS, Pen/Strep, glutamine) was added to the cells and incubated for 48 h. Cell monolayers were fixed (0.8% glutaraldehyde, 2.2% formaldehyde in DPBS) for 8 min and stained for β-galactosidase expression (4 mM potassium ferricyanide, 4 mM potassium ferrocyanide, 400 µg/ml magnesium chloride, 400 µg/ml X-gal in DPBS) for 2 h. Titer (2.5 × 10^7^ PFU/ml) was calculated by counting “Blue” β-gal expressing cells.

### Innate immune stimulations

For innate immune stimulation studies, cells were seeded at 5 × 10^5^ cells/ml in complete RPMI in 12-well plates and cultured overnight at 37 °C. The following day cells were treated with media containing human recombinant IFNβ (Toray Industries), PMA (Sigma), ionomycin, or Sendai virus (SeV) in amounts described in Fig. legends. At time points indicated in Fig. legends, cells were collected and lysates harvested for protein or RNA analysis as described below.

### RNA synthesis and transfection

HCV Con1 nonstimulatory RNA (xRNA) and HCV Con1 pU/UC RNA (PAMP RNA) were synthesized from T7 promoter-linked complementary oligonucleotides (Integrated DNA Technologies). All in vitro-transcribed RNAs contained a 5' triphosphate (5'-ppp) and three guanine nucleotides at the 5' end to enhance T7 polymerase transcription. RNA products were generated using T7 RNA polymerase and a T7 MEGAshortscript kit (Ambion) according to the manufacturer's instructions. Following in vitro transcription, DNA templates were removed with DNase treatment and unincorporated nucleotides and protein were removed from the reaction mixture by phenol–chloroform extraction. RNA was then precipitated using ethanol and ammonium acetate as described by the manufacturer and resuspended in nuclease-free water. RNA concentrations were determined by absorbance using a Nanodrop spectrophotometer. RNA quality was assessed on denaturing 2% agarose formaldehyde gels [78]. For RNA transfection experiments, cells were seeded at 5 × 10^5^ cells/ml in 12-well plates and cultured overnight at 37 °C. Transfections were performed using 10 pmol RNA with a TransIT-mRNA transfection kit (Mirus) according to the manufacturer's instructions.

### qRT-PCR

For quantification of HIV RNA or host gene expression, Cell lysates were digested in RLT and total cellular RNA extracted using the miRNeasy Micro kit (Qiagen) or miRNeasy Mini kit (Qiagen) according to the manufacturer's instructions, and residual genomic DNA removed by DNAse treatment. RNA concentrations were determined by absorbance using the NanoDrop 2000 Spectrophotometer. cDNA was synthesized using the iScript Select cDNA Synthesis kit (Biorad). Quantitative real-time PCR (qRT-PCR) was performed using SYBR Green PCR master mix (ABI) and primers (see Additional file 1: Table S4), on a ViiA 7 Real-Time PCR System (Applied Biosystems) with QuantStudio software (Applied Biosystems).

For all qRT-PCR analysis, ΔΔCt values were calculated relative to housekeeping gene *RPL13A* and to control (untreated or mock-infected) cells as described in figure legends. Mean fold change (FC) was calculated relative to control cells as indicated in figure legends. Multiple independent experiments were performed for each study, and presented data represent mean FC ± SD of biological replicates from multiple combined independent experiments or from a single representative experiment as indicated in figure legends.

### Immunoblotting

Whole cell lysates were prepared with RIPA buffer (25 mM Tris, HCl pH 7.6, 150 mM NaCl, 1% NP-40, 1% sodium deoxycholate, 0.1% SDS) supplemented with 1% protease inhibitor, 1% phosphatase inhibitor, and 0.1% okadaic acid. Ten micrograms of protein were loaded in equal volumes and separated on a 4–20% Mini-Protean TGX precast gel (Biorad), then transferred to a nitrocellulose membrane and blocked for 1 h at room temp with 5% BSA (Sigma) in TBST. Blots were incubated overnight at 4 °C with primary antibodies from Cell Signaling Technology: rabbit (Rb) anti-MX1, Rb anti-OAS1, Rb anti-phospho-STAT1 Y701, Rb anti-STAT1, Rb anti-STAT2, Rb anti-phospho-STAT3 S727, or Rb anti-STAT3, Rb anti-phospho-IRF3 S386. The following primary antibodies were also used: Rb anti-MX2 (Novus Bio), Rb anti-IFIT1 (gift from G. Sen at the Cleveland Clinic), mouse (Ms) anti-IFITM1 (ProteinTech), Ms anti-Actin (Sigma), Rb anti phospho-JAK1 Y1022/1023 (Abcam), Rb anti phospho-STAT2 Y689 (Millipore), and Ms anti-IRF3 clone AR1 [79]. Following primary antibody incubation, blots were washed with TBST then incubated with the appropriate horseradish peroxidase (HRP)-conjugated secondary antibody (Jackson Immunoresearch Labs) for 1 h at room temp. Blots were washed in TBST then target proteins detected with a chemiluminescent kit (ThermoFisher) and imaged using a ChemiDoc XRS + system (Bio-Rad) with Image Lab Software (Bio-Rad). Target protein abundance relative to actin was quantified using ImageJ software.

### Flow cytometry analysis

IFNAR surface expression on unstimulated cell lines was measured by flow cytometry. Cells were collected in eppendorf tubes, washed once in cold FACS buffer (PBS with 2% FBS and 0.02% sodium azide), then stained for 30 min with mouse anti-IFNAR 4G8 antibody (Sigma) or purified mouse IgG2a K isotype control (BioLegend). Cells were washed twice with FACS buffer then incubated for 30 min with rat anti-mouse IgK light chain APC-Cy7 secondary antibody (BD Biosciences). Cells were washed twice with cold FACS buffer then stained with 0.05 μg/ml DAPI (ThermoFisher) for 10 min. Cells were washed three times in DPBS then resuspended in FACS buffer. Data were acquired on a Canto RUO cytometer (BD Biosciences) and analyzed using FlowJo software (FlowJo LLC).

### ImageStream analysis of IRF3 nuclear translocation

Following Sendai virus infection (100 HAU/ml, 24 h), IRF3 nuclear localization was measured in cell lines by ImageStreamX technology as described elsewhere [79]. Briefly, cells were washed in PBS, fixed and permeabilized using the BD Cytofix/Cytoperm kit per the manufacturer's instructions (BD Biosciences), then stained with mouse monoclonal anti-IRF3 AR1 antibody on ice for 30 min. Cells were washed twice with PBS + 0.1% sodium azide, then stained with AF647 anti-mouse secondary antibody on ice for 30 min. Cells were washed with PBS + 0.1% sodium azide, stained with 0.05 μg/ml DAPI (ThermoFisher) for 10 min, washed twice with PBS + 0.1% sodium azide, then resuspended in 50 μl Perm/Wash (BD Biosciences) for analysis by an Annis ImageStreamX Mk II imaging cytometer. IRF3 expression and DAPI nuclei were measured in 20,000 cells per sample, and expression similarity above an arbitrary cutoff determined using IDEAS software (Luminex) as shown in Supplemental Fig. 2a.

### Bulk RNA-seq

Jurkat and JLat9.2 cells were treated with cell culture media for 4 h (mock) or 100 IU/ml IFNβ for 4, 8, or 12 h as described in figure legends. Cells were digested in RLT buffer then RNA extracted using a miRNeasy Micro kit (Qiagen) as described above. The quality and concentration of the recovered RNA was determined using a LabChip GXII (PerkinElmer) instrument and a ribogreen-based RNA assay, respectively. mRNA-seq libraries were constructed using KAPA Stranded mRNA-Seq Kit (Roche, Indianapolis, IN) following the manufacturer's recommended protocol. Libraries were sequenced on an Illumina NextSeq500 sequencer using Illumina NextSeq 500/550 High Output v2 kits (150 cycles) following the manufacturer's protocol for sample handling and loading. Sequencing run metrics were visualized for quality assurance using Illumina's BaseSpace platform, and the quality of mRNA-seq reads were assessed using FastQC version 0.11.3 (http://www.bioinformatics.babraham.ac.uk/projects/fastqc). Adapters were digitally removed using cutadapt, version 1.8.3 [80]. Subsequently, raw RNA-seq reads were demultiplexed, checked for quality with FastQC v0.11.5 [81], and ribosomal RNA reads were digitally removed with bowtie2 v2.3.4 [82]. Reads were then mapped to the human genome (GRCh37) with STAR v2.4.0h1 [83], resulting in at least 20 million uniquely mapped reads per sample. Gene counts were quantified with htseq-count v 0.6.1p1 [84] specifying –stranded = reverse and –mode = intersection-nonempty. Gene counts were loaded into the R statistical programming language (v4.0.0, R Core Team 2020) using RStudio v1.2.1335. We first removed genes with less than 10 raw counts averaged across all samples leaving 13,849 genes for analyses. Counts were then normalized via trimmed mean of M values implemented in edgeR and transformed into log2 counts per million with the voom function of the limma package [85, 86]. Samples then underwent principal component analysis and results were visualized with ggplot2 v3.3.2 [87]. All differential expression analyses were performed with limma. To assess functional differences between Jurkat and JLat9.2 mock samples, were performed gene set enrichment analysis (GSEA) using WebGestaltR [88]. Genes were ranked by descending t-statistic values calculated in limma and were tested for enrichment among KEGG pathways and the non-redundant Gene Ontology database. We designed contrast matrices to test for the effect of IFNβ treatment in each cell line over time relative to mock (e.g. Jurkat IFNβ 4 h – Jurkat Mock 4 h) and to directly compare cell lines while accounting for baseline differences (e.g. [(Jurkat IFNβ 4 h – Jurkat Mock 4 h) – (JLat9.2 IFNβ 4 h – JLat9.2 Mock 4 h)]). A gene was considered differentially expressed if the absolute Fold Change > 1.5 and Benjamini-Hochberg-adjusted p-value < 0.05 in at least one comparison [89]. The union of all differentially expressed genes were grouped into co-expression modules (ward.D clustering, Euclidean distance) with WGCNA [90, 91] and gplots heatmap.2. These modules were uploaded into Ingenuity Pathway Analysis software [92] to assess pathway enrichment and upstream regulators among co-expressed genes.

### RGH virus infection and FACS sorting

Jurkat cells were seeded at a concentration of 5 × 10^5^ cells in 6 well plates in complete RPMI supplemented with 4 μg/ml polybrene, and spinoculated with HEK-293 T conditioned media (mock) or VSV-pseudotyped RGH viral stocks (MOI 0.2) for 1.5 h at 500×G. Cells were cultured for 24 h, washed twice with DPBS, then cultured in complete RPMI for an additional 4 days to allow establishment of latency. Infected cells were washed in FACS buffer (DPBS + 2% FBS + 0.02% sodium azide), stained with 0.05 μg/ml DAPI (ThermoFisher) to identify dead cells, and viably sorted based on mCherry and GFP expression on a FACSAria II cytometer (BD Biosciences). Sorted cells were resuspended in complete RPMI, seeded at 3 × 10^5^ cells/ml in 0.5 ml in 12-well plates, cultured overnight, then stimulated for 8 h with 100 IU/ml IFNβ or reactivated for 24 h with 10 nM PMA and 1 μM ionomycin.

### NanoLuc HIV infection and ART suppression of primary CD4 + T cells

Purified CD4 + T cells were thawed and cultured at 10^6^ cells/ml for 5 days in complete RPMI media supplemented with cytokines that aid in homeostatic proliferation and HIV susceptibility: 20 U/ml IL-2, 10 ng/ml IL-7, and 50 ng/ml recombinant human IL-15 (all from Peprotech). CD4 + T cells were then seeded at a concentration of 2 × 10^6^ cells/ml in 6-well plates and were spinoculated with complete RPMI media (mock control) or NanoLuc HIV (MOI 1.0 or 2.0, see figure legends) for 2 h at 2400 rpm. Cells were washed three times with RPMI and cultured in T25 flasks for 24 h in complete RPMI supplemented 20 U/ml IL-2, 10 ng/ml IL-7, and 50 ng/ml IL-15. HIV replication was then suppressed for 7 days by treatment with ART consisting of 10 μM Raltegravir (NIH AIDS Reagent Program) and 1 μM Efavirenz (NIH AIDS Reagent Program). During the week of ART suppression, cells were washed and resuspended in fresh medium, cytokines, and ART every two days. Aliquots of supernatants were withdrawn and frozen throughout the ART time course to monitor ART suppression. To determine percent of infected cells, genomic DNA was isolated from cells before ART (24 h post infection) and after ART (8 days post infection), and number of HIV copies/cell measured by ddPCR (Additional file 1: Table S3). At 8 days post infection (dpi), suppressed cells were seeded in 12-well plates at 5 × 10^5^ cells/ml in complete RPMI + cytokines ± ART, and were treated with or without 100 IU/ml IFNβ. At 8 h following IFNβ treatment, cells were fixed in methanol for single cell sequencing analysis, or lysates collected for qRT-PCR or Immunoblot analysis. For reactivation studies at 8 dpi, suppressed cells were seeded in 12-well plates at 5 × 10^5^ cells/ml in complete RPMI + cytokines ± ART, and were treated for 24 h with 10 nM PMA and 1 μM ionomycin, then lysates collected for qRT-PCR analysis.

### Luciferase reporter assay

Samples were brought to room temperature and an aliquot of 20 µl of each sample was mixed with 20 µl of 1X Nano-Glo® luciferase assay reagent (Nano-Glo® Luciferase Assay System, Promega) in a white flat-bottom polystyrene 96-well plate (Corning, Sigma-Aldrich). The mixtures were incubated for 10 min in the dark and luminescence was read on an MLX 96 Well Plate Luminometer (1 s/well, read height: 1 mm, Dynex Technologies) and reported in relative light units (RLU).

### Droplet digital PCR (ddPCR) analysis of HIV-1 proviral DNA

To quantify the number of HIV genomes per infected cell, we used a droplet digital PCR (ddPCR) assay detecting the HIV gag gene. Genomic DNA was purified from frozen cellular pellets using PureLinkTM Genomic DNA Mini Kit (Thermo Fisher Scientific), following manufacturer’s instructions. The concentration of DNA of each sample was determined using NanoDrop™ One-W (Thermo Scientific). Five µl of undiluted and/or 1:10 diluted gDNA were used per reaction. To normalize for total genomic DNA and estimate the number of cells per reaction, we quantified copy numbers of the cellular peptidylprolyl isomerase A (PPIA) gene using a specific FAM-conjugated primers/probe assay (cyclophilin A; assay ID: Hs.571 PT.58v.38887593.g; FAM/ZEN/3IABkFQ configuration). For amplification of the HIV gag region, we used the following primers and probe: Gag sense 5 ‘GACTAGCGGAGGCTAGAAGGAGAGA 3 ‘; Gag antisense 5 ‘ CTAATTCTCCCCCGCTTAATAYTGACG 3 ‘; Gag probe 5 ‘ HEX AT + G + GGT + GC + GAGA/3BHQ_1 3 ‘, wherein “ + ” indicates locked nucleic acids and BHQ denotes 3’ Black quencher©-1 [93].

The ddPCR reaction was done in a total volume of 22 µl of a mixture containing 11 µl of 2X ddPCR Supermix for Probes (BioRad), 1.1 µl 20X hexachlorofluorescein (HEX) HIV gag specific Taqman assay (Integrated DNA Technologies), 1.1 µl of 20X 6-carboxyfluorescein (FAM)-labeled PPIA target qPCR assay and genomic DNA. Each assembled ddPCR reaction mixture was loaded in duplicate into the wells of an eight-channel disposable droplet generator cartridge (BioRad) and droplet generation oil (BioRad) was added. After droplet generation, the samples were amplified to endpoint in 96-well PCR plates on a conventional thermal cycler (C1000, Biorad) using the following conditions: denaturation/enzyme activation for 10 min at 95 °C, 40 cycles of 30 s denaturation at 94 °C and 60 s annealing/amplification at 60 °C, followed by a final 10 min incubation step at 98 °C. After PCR, the droplets were read on the QX100 Droplet Reader (BioRad). ddPCR data analysis was performed with QuantaSoft analysis software version 1.3.1.0 (BioRad). A non-template control well containing ddPCR reaction mix but no cDNA and a mock-infected control were included to adjust the reaction threshold.

### Single cell RNA-seq: cell preparation

Purified CD4 + T cells were thawed, activated with cytokines, infected with NanoLuc HIV, suppressed with ART for 7 days, then stimulated with 100 IU/ml IFNβ for 8 h as described above. At 8 h post IFNβ stimulation, cells were washed twice with cold PBS then viability assessed by Trypan Blue exclusion. Viable cell suspension was adjusted to 5 × 10^6^ cells/ml in 200 μl cold PBS, then 800 μl cold methanol slowly added. Methanol-fixed cells were stored at − 20 °C until further sample processing. After two weeks, fixed cells were thawed, centrifuged and supernatant removed, then resuspended in rehydration buffer (SSC buffer supplemented with 1% BSA, 20 U/μl Superasein, and 1 M DTT). Single-cell suspensions were diluted to a cell concentration of 1,000 cells/µl for single-cell RNAseq.

### Single cell RNA-seq: library preparation and sequencing

Single-cell RNA sequencing was performed according to the manufacturer’s instructions (10 × Genomics). Single-cell suspensions were loaded onto a Chromium Single Cell Chip G at a target capture rate of ~ 8000 individual cells per sample. Barcoded, full-length cDNAs from poly-adenylated mRNAs were produced following reverse transcription of the resulting Gel Beads-in-emulsion (GEMs). All samples for a given donor were processed simultaneously with the Chromium Controller (10 × Genomics) to create purified cDNA. Indexed libraries were prepared from 10 µl of cDNA using Chromium Next GEM Single Cell 3’ Reagent Kits v3 (10 × Genomics). Library and cDNA quality were evaluated using the Agilent 2100 Bioanalyzer Instrument (Agilent) and quantified using the ViiA 7 Real-Time PCR system (ThermoFisher). Constructed libraries were sequenced on an Illumina NextSeq 500/550 High Output v2 kit (Illumina). Each sample was sequenced to a depth of 40,000 reads per cell.

### Single cell RNA-seq: data processing and analysis

Single cell reads were aligned to the hg19 genome with CellRanger v4.0.0 [94]. In the hg19 genome we integrated the HIV-1 vector PNL4-3 (GenBank AF324493.2) and counted reads that map to HIV. We used Seurat 3.2.3 to normalize, perform dimensionality reduction (UMAPs), and identify differentially expressed genes [95]. Using Seurat we filtered out cells from the analysis that had less than 200 or greater than 2,500 genes detected and a mitochondrial DNA percentage greater than 10%. Cells were classified as HIV + if they had at least one read mapping to the HIV genome (one UMI count). Differential gene analyses were performed through Seurat using MAST [96, 97] and significant differentially expressed genes had an absolute log fold change of ≧ 1.2 and an adjusted P value of less than 0.05. Over-representation analysis using non-redundant biological process gene-ontology terms on viral induced genes (Fig. 6c) was performed using the tool Web-based Gene Set Analysis Toolkit (http://www.webgestalt.org). Cells were classified into T cell subtypes using Monocle3/Garnett [98] using the markers listed in Additional file 1: Figure S7b. Linear regression models were performed using the lm() function within R and plotted with ggplot2. Code for this analysis is available at: https://github.com/galelab/Olson_Latent_HIV_Infection.

### Quantification and statistical analysis

For qRT-PCR analyses and protein abundance quantification, statistical tests were performed using Prism 8.0 software (GraphPad). Data are presented as the values ± standard deviation (SD). Statistical significance was determined using a two-tailed Student's t test with multiple comparisons (Holm-Sidak post-test) or by two-way ANOVA with multiple comparisons (Holm-Sidak post-test) as indicated in figure legends. For these tests, p < 0.05 was considered statistically significant. *p < 0.05, **p < 0.01, ***p < 0.001. For bulk RNA-seq and scRNA-seq analysis, statistical analysis was performed using RStudio v1.2.1335 (R Core Team) as described in corresponding methods sections. In this study, n is defined as the number of independent, non-technical biological replicates within a single representative experiment, or as the number of independent experiments included in a data set, as detailed in figure legends.

## Supplementary Information

**Additional file 1: Figure S1**. Analysis of latent cell line response to IFNβ stimulation. a,b qRT-PCR analysis of ISG mRNA expression in resting Jurkat vs JLat11.1 cells (a) or following treatment with 100 IU/ml IFNβ for indicated times (b). Fold change (FC) was calculated relative to untreated Jurkat cells (ΔΔCt method), and each symbol represents mean FC + SD of three technical replicates from a single experiment. Data from Jurkat cells are also shown in Fig. 1c & e. Statistical significance relative to similarly treated control Jurkat cells was calculated by unpaired Student’s t-test; asterisks denote significance (*p<0.05, **p<0.01, ***p<0.001). c-d qRT-PCR analysis of HIV RNA expression in Jurkat vs JLat9.2 (c) cells or A3.01 vs ACH2 cells (d) following treatment with 100 IU/ml IFNβ for indicated times. e FACS analysis of percent of JLat9.2 cells expressing GFP (indicating HIV reactivation) after mock treatment (culture media alone), reactivation with 8nM PMA for 24h, or infection with 100 HAU/ml Sendai virus (SeV) for 24h. f-h FACS analysis of IFNAR1 surface expression compared to isotype control in Jurkat vs JLat9.2, Jurkat vs JLat11.1, or A3.01 vs ACH2 cells. i,j ImageJ quantification of target protein abundance from immunoblots of Jurkat or JLat9.2 cells stimulated with 100 IU/ml IFNβ for the time points indicated (See Fig. 1g & h). One experiment was performed in Fig. S1i and three independent experiments were performed in S1j. Values represent mean ± SD expression ratio over actin. In panel j, statistical significance in latent cell lines relative to uninfected, untreated control cell lines was determined by unpaired Student’s t-test; asterisks denote significance (*p<0.05, **p<0.01, ***p<0.001). **Figure S2**. Flow cytometry gating schemes for Jurkat cell models of latency. a Gating scheme used to determine IRF3 nuclear localization by ImageStreamX technology in mock-infected (media) or SeV-infected (100 HAU/ml, 24h) Jurkat or JLat9.2 cells. Cells with an IRF3/DAPI similarity value over the arbitrary cutoff 2.3 are determined positive for IRF3 nuclear translocation (See Fig. 2f-g). Data in (a) are from SeV-infected Jurkat cells. b Gating scheme used to sort Jurkat cells after infection with RGH virus (5d, MOI 0.2). Dead cells were excluded by DAPI staining, and infection groups sorted based on mCherry and GFP expression. Productive infection: mCherry+GFP+; latent infection: mCherry+GFP-; no infection: mCherry-GFP-. **Figure S3**. RNA seq analysis of Jurkat and JLat9.2 cells after IFN stimulation. a Principal component analysis (PCA) clusters Jurkat and JLat9.2 cells by treatment condition: mock treatment (media, 4h) or IFNβ stimulation (100 IU/ml for 4h, 8h, or 12h). One experiment was performed with three biological replicates per treatment condition. Each data point represents a biological replicate (n = 3) for each treatment condition. b Network analysis showing select STAT1- and STAT2-dependent genes identified in differential of differential expression (DDE) analysis (see Fig. 3). 106 DDE genes were identified to have significantly different induction by IFN in Jurkat relative to JLat9.2 cells. Genes are colored according to log2 FC of differential expression of IFNstimulated genes in JLat9.2 relative to Jurkat cells. Red: greater induction by IFN in JLat9.2 compared to Jurkat cells; Blue: greater induction by IFN in Jurkat cells compared to JLat9.2 cells. **Figure S4**. Luciferase and ISG expression analysis in a primary CD4+ T cell model of HIV suppression Primary CD4+ T cells from three healthy human donors were cultured for 5 days in homeostatic cytokines (IL-2, IL-7, IL-15) then mockinfected (media) or infected with NanoLuc HIV at MOI 2.0 (Donor 1 & 2) or MOI 1.0 (Donor 3) for 24h. Viral replication was suppressed for 7 days with ART (10 μm raltegravir and 1 μm efavirenz), then at 8 days post infection (dpi) cells were stimulated with various agonists as detailed below. a qRT-PCR analysis of HIV RNA from HIV-infected cells 24h after infection, prior to ART. No HIV RNA was detected in mockinfected samples. Bars represent HIV RNA expression relative to an arbitrary Ct value of 40 from one representative experiment. b Supernatant was collected from mock or HIV-infected samples at indicated time points and analyzed for luciferase expression indicating HIV transcription. Data points represent 12 technical replicates collected from each sample in one representative experiment (see also Fig. 5C). c qRT-PCR analysis of baseline ISG expression in HIV-infected cells after 7 days of ART suppression (8 days post infection). Bars represent mean FC ± SD relative to mock-infected control for each donor. Data represents three biological replicates per treatment condition. Statistical significance relative to mock-infected control was determined by two-tailed t-test (Holm-Sidak). d Luciferase expression analysis of supernatant from mock-infected or HIV-infected samples that were stimulated at 8 dpi with IFNβ (100 IU/ml, 8h). e Immunoblot analysis of mock-infected vs HIV-infected CD4+ T cells that were stimulated at 8 dpi with IFNβ (100 IU/ml) for the indicated times (Donor #2 only). f Luciferase expression analysis of supernatant from mock-infected or HIV-infected samples that at 8 dpi were cultured with or without ART (24h), and with or without PMA/ionomycin (16nM/1μM, 24h). For Panels d & f, bars represent mean FC + SD luciferase readings of three biological replicates from one experiment for each donor. Statistical significance of IFN-treated relative to untreated cells within each infection group (d) or relative to indicated HIV-infected control (f) was calculated by two-way ANOVA with multiple comparisons (Holm-Sidak). For all data in this figure, multiple independent experiments were performed and data is shown from one representative experiment. For all statistical tests: *p<0.05, **p<0.01, ***p<0.001. **Figure S5**. scRNA-seq analysis of T cell subsets. Primary CD4+ T cells from two healthy human donors were cultured for 5 days in homeostatic cytokines (IL-2, IL-7, IL-15) then mock-infected (media) or infected with NanoLuc HIV at MOI 2.0 for 24h. Viral replication was then suppressed for 7 days with ART (10 μm raltegravir and 1 μm efavirenz). At day 8 post infection, cells were stimulated for 8h with IFNβ (0, 20, or 100 IU/ml) then analyzed by single cell RNA sequencing (scRNA-seq). a,b scRNA-seq analysis of T cell identity per sample using the markers listed on the right. c Percent of vRNA+ cells within each T cell subset for each HIV-infected sample. **Figure S6**. scRNA-seq analysis of ISG average expression across all primary CD4+ T cell samples. Heat map showing single cell RNA sequencing (scRNA-seq) analysis of average expression of 116 ISGs in all samples tested. CD4+ T cells from two healthy human donors (Donor 1 & 2) were cultured for 5 days in homeostatic cytokines (IL-2, IL-7, IL-15), then mock-infected (media) or HIV-infected (Nanoluc HIV, MOI 2.0). 24h after infection cells were suppressed with ART for 7 days, then stimulated with IFNβ (100 IU/ml, 8h), and analyzed by scRNA-seq. Each pixel column is the expression of an individual cell. Brackets denote genes significant in each comparison as described in Fig. 7a. **Table S3**. HIV expression in primary CD4+ T cells. **Table S4**. Oligonucleotides used in this study.

**Additional file 2: Table S1A**. Significant DE genes identified in IFN-treated Jurkat and/or JLat cells (corresponds to Fig. 3B). S1B. Significantly enriched pathways with associated DE genes (corresponds to colored modules on heat map in Fig. 3B). S1C. Significant DDE genes differentially induced by IFN in Jlat9.2 vs Jurkat cells (corresponds to Fig. 3C).

**Additional file 3: Table S2A**. HIV regulated genes identified in differential expression analysis of vRNAhi & vRNAlo cell subsets relative to mock (corresponds to Fig. 6D). S2B. Significant DE genes in vRNAhi cells relative to mock (corresponds to Fig. 6D). S2C. Significant DE genes in vRNAlo cells relative to mock (corresponds to Fig. 6D). S2D. Average expression of ISGs in HIV-infected cell subsets (vRNAhi, vRNAlo, vRNA-) and mock-infected cells, with or without IFN (corresponds to FIG S5C). S2E. Log2 fold change gene expression values of downregulated genes in vRNAhi, vRNAlo, or vRNA- cells (corresponds to Figs. 7b-d).

## Data Availability

The datasets analyzed during the current study are available from the corresponding author on reasonable request. Transcriptomic data sets are available through the Gene Expression Omnibus (https://www.ncbi.nlm.nih.gov/geo/) under access numbers GSE174380 (bulk RNA sequencing) and GSE176386 (single cell RNA sequencing). Code for the single cell RNA sequencing analysis is available at: https://github.com/galelab/Olson_Latent_HIV_Infection.

## Abbreviations

ART: Antiretroviral therapy
CMV: Cytomegalovirus
DDE: Differential of differential expression
ddPCR: Droplet digital polymerase chain reaction
DE: Differential expression
EMCV: Encephalomyocarditis virus
GO: Gene ontology
HIV: Human immunodeficiency virus 1
IFN: Interferon
IRF3: Interferon regulatory factor 3
ISG: Interferon-stimulated gene
LTR: Long terminal repeat
NanoLuc HIV: Secreted nanoluciferase reporter human immunodeficiency virus 1
PAMP: Pathogen-associated molecular pattern
PRR: Pathogen recognition receptor
RGH: Red-green HIV-1
RNA-seq: RNA sequencing
ScRNA-seq: Single cell RNA sequencing
SeV: Sendai virus
SIV: Simian immunodeficiency virus
VSV: Vesicular stomatitis virus
