# Supplementary material for: Innate immune regulation in HIV latency models
Source: Retrovirology. 2022 Jul 8;19:15. doi: 10.1186/s12977-022-00599-z (PMC9270781; doi:10.1186/s12977-022-00599-z)
Supplement: Supplementary file 1 — Additional file 1: Figure S1. Analysis of latent cell line response to IFNβ stimulation. a,b qRT-PCR analysis of ISG mRNA expression in resting Jurkat vs JLat11.1 cells (a) or following treatment with 100 IU/ml IFNβ for indicated times (b). Fold change (FC) was calculated relative to untreated Jurkat cells (ΔΔCt method), and each symbol represents mean FC + SD of three technical replicates from a single experiment. Data from Jurkat cells are also shown in Fig. 1c & e. Statistical significance relative to similarly treated control Jurkat cells was calculated by unpaired Student’s t-test; asterisks denote significance (*p<0.05, **p<0.01, ***p<0.001). c-d qRT-PCR analysis of HIV RNA expression in Jurkat vs JLat9.2 (c) cells or A3.01 vs ACH2 cells (d) following treatment with 100 IU/ml IFNβ for indicated times. e FACS analysis of percent of JLat9.2 cells expressing GFP (indicating HIV reactivation) after mock treatment (culture media alone), reactivation with 8nM PMA for 24h, or infection with 100 HAU/ml Sendai virus (SeV) for 24h. f-h FACS analysis of IFNAR1 surface expression compared to isotype control in Jurkat vs JLat9.2, Jurkat vs JLat11.1, or A3.01 vs ACH2 cells. i,j ImageJ quantification of target protein abundance from immunoblots of Jurkat or JLat9.2 cells stimulated with 100 IU/ml IFNβ for the time points indicated (See Fig. 1g & h). One experiment was performed in Fig. S1i and three independent experiments were performed in S1j. Values represent mean ± SD expression ratio over actin. In panel j, statistical significance in latent cell lines relative to uninfected, untreated control cell lines was determined by unpaired Student’s t-test; asterisks denote significance (*p<0.05, **p<0.01, ***p<0.001). Figure S2. Flow cytometry gating schemes for Jurkat cell models of latency. a Gating scheme used to determine IRF3 nuclear localization by ImageStreamX technology in mock-infected (media) or SeV-infected (100 HAU/ml, 24h) Jurkat or JLat9.2 cells. Cells with an IRF [file 12977_2022_599_MOESM1_ESM.pdf]

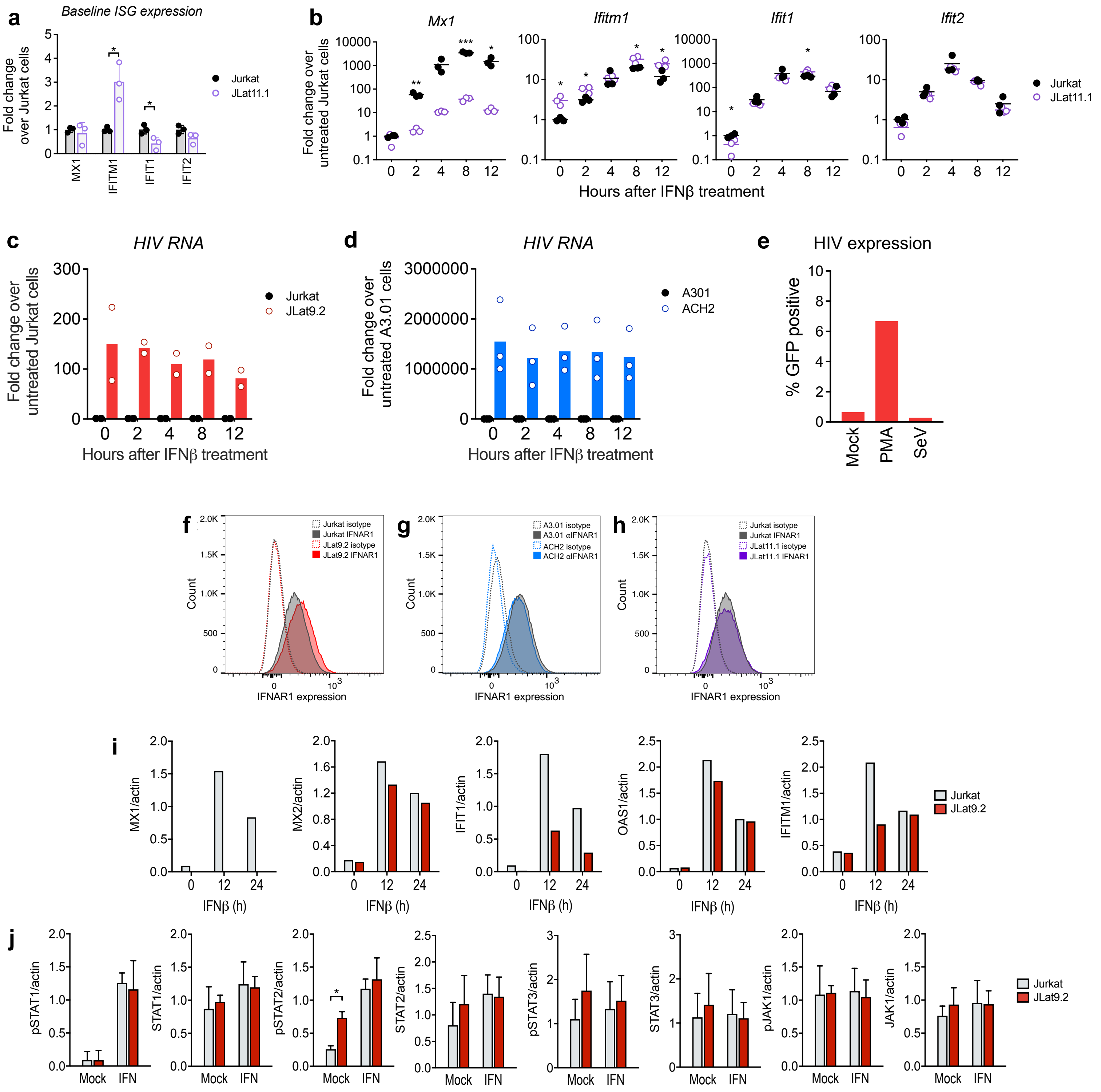

Supplemental Figure 1. Analysis of latent cell line response to IFNβ stimulation

a,b qRT-PCR analysis of ISG mRNA expression in resting Jurkat vs JLat11.1 cells (a) or following treatment with 100 IU/ml IFNβ for indicated times (b)**.** Fold change (FC) was calculated relative to untreated Jurkat cells (ΔΔCt method), and each symbol represents mean FC + SD of three technical replicates from a single experiment. Data from Jurkat cells are also shown in Figs. 1c & 1e. Statistical significance relative to similarly treated control Jurkat cells was calculated by unpaired Student's t-test; asterisks denote significance (*$p<0.05$, **$p<0.01$, ***$p<0.001$). **c-d** qRT-PCR analysis of HIV RNA expression in Jurkat vs JLat9.2 (c) cells or A3.01 vs ACH2 cells (d) following treatment with 100 IU/ml IFNβ for indicated times. **e** FACS analysis of percent of JLat9.2 cells expressing GFP (indicating HIV reactivation) after mock treatment (culture media alone), reactivation with 8nM PMA for 24h, or infection with 100 HAU/ml Sendai virus (SeV) for 24h. **f-h** FACS analysis of IFNAR1 surface expression compared to isotype control in Jurkat vs JLat9.2, Jurkat vs JLat11.1, or A3.01 vs ACH2 cells. **i,j** ImageJ quantification of target protein abundance from immunoblots of Jurkat or JLat9.2 cells stimulated with 100 IU/ml IFNβ for the time points indicated (See Figs. 1g & 1h). One experiment was performed in Fig. S1i and three independent experiments were performed in S1j. Values represent mean ± SD expression ratio over actin. In panel j, statistical significance in latent cell lines relative to uninfected, untreated control cell lines was determined by unpaired Student's t-test; asterisks denote significance (*$p<0.05$, **$p<0.01$, ***$p<0.001$).

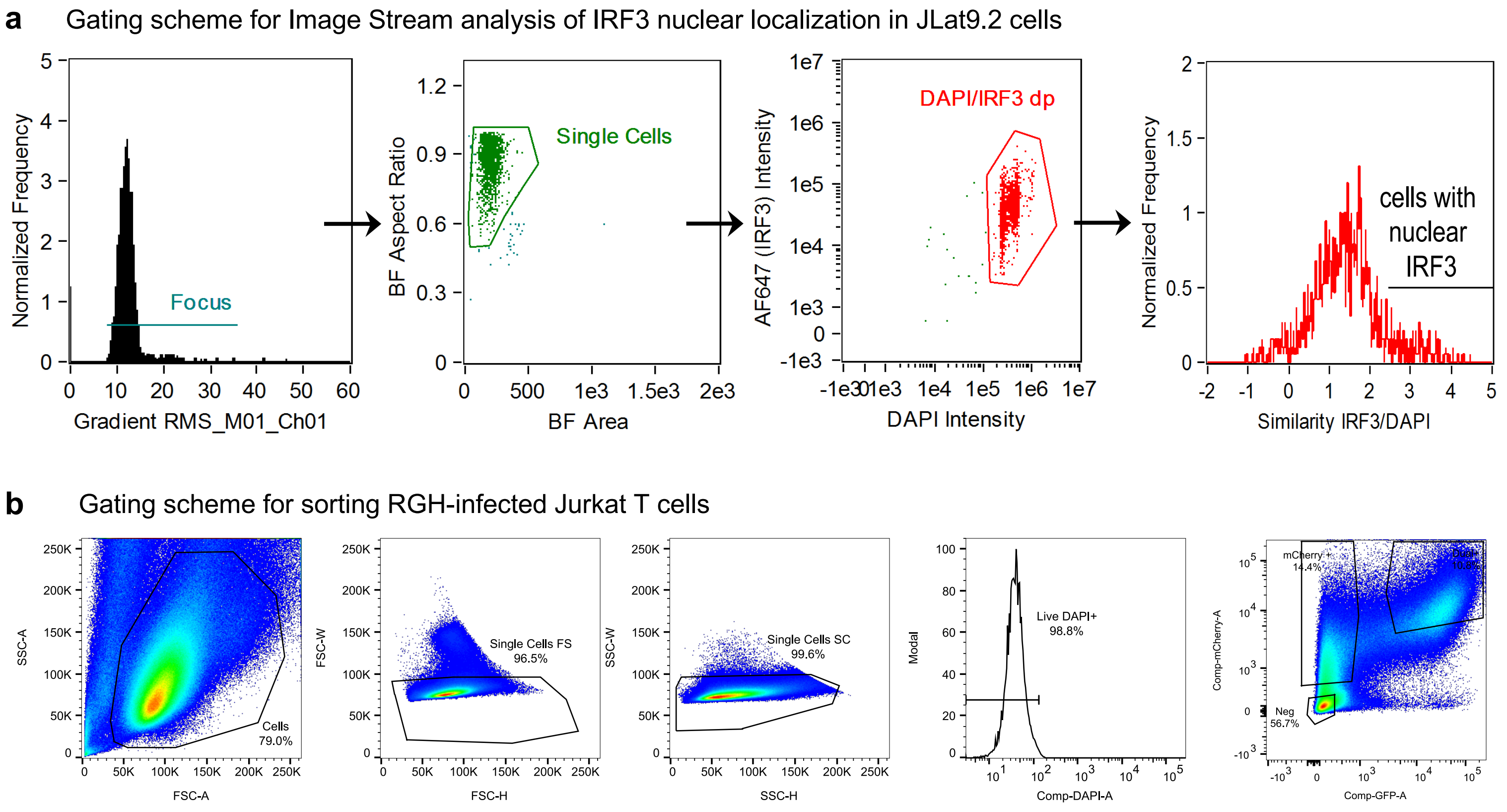

Supplemental Figure 2. Flow cytometry gating schemes for Jurkat cell models of latency

a Gating scheme used to determine IRF3 nuclear localization by ImageStreamX technology in mock-infected (media) or SeV-infected (100 HAU/ml, 24h) Jurkat or JLat9.2 cells. Cells with an IRF3/DAPI similarity value over the arbitrary cutoff 2.3 are determined positive for IRF3 nuclear translocation (See Figs. 2f-g). Data in (a) are from SeV-infected Jurkat cells. **b** Gating scheme used to sort Jurkat cells after infection with RGH virus (5d, MOI 0.2). Dead cells were excluded by DAPI staining, and infection groups sorted based on mCherry and GFP expression. Productive infection: mCherry+GFP+; latent infection: mCherry+GFP-; no infection: mCherry-GFP-.

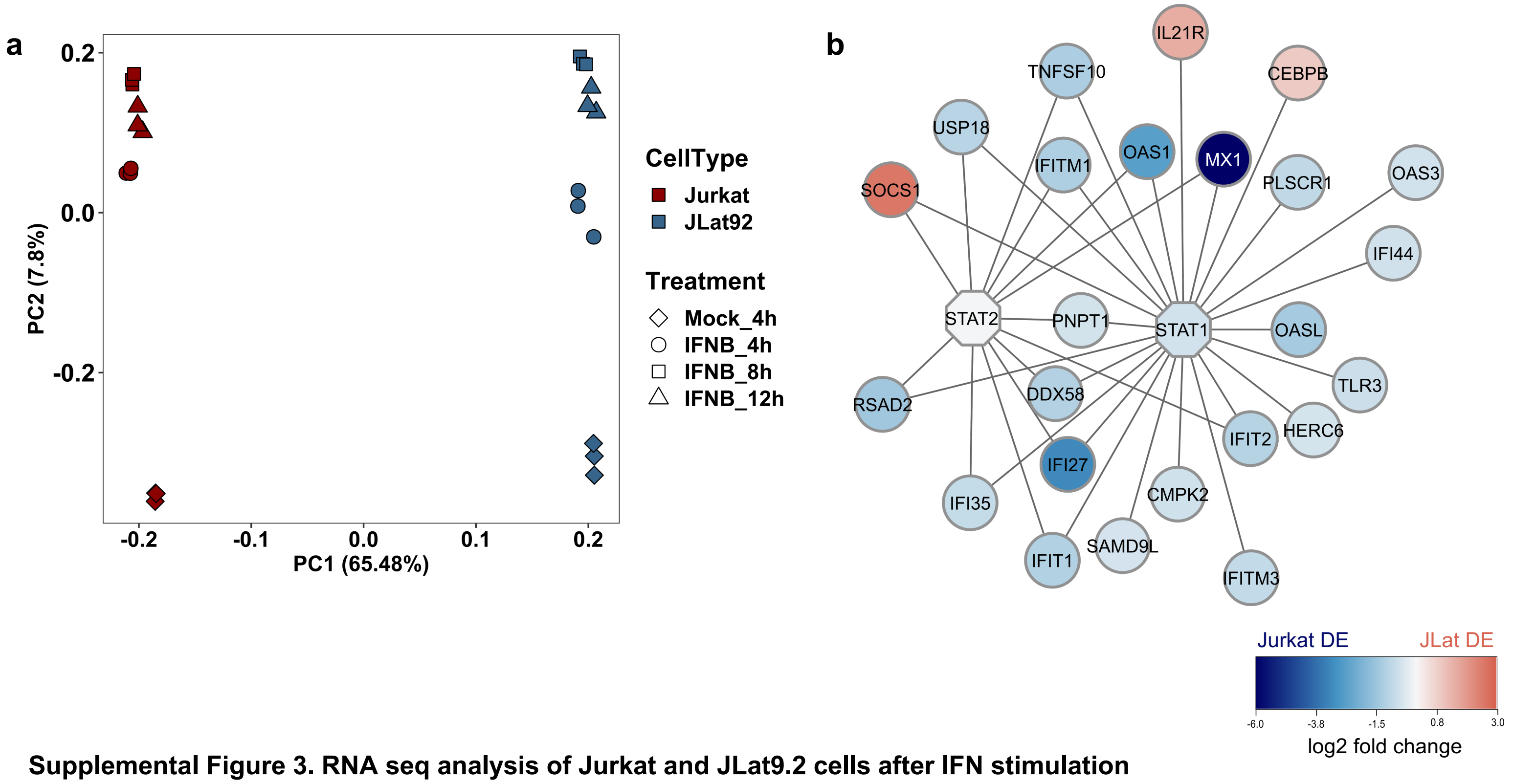

Supplemental Figure 3. RNA seq analysis of Jurkat and JLat9.2 cells after IFN stimulation

a Principal component analysis (PCA) clusters Jurkat and JLat9.2 cells by treatment condition: mock treatment (media, 4h) or IFNβ stimulation (100 IU/ml for 4h, 8h, or 12h). One experiment was performed with three biological replicates per treatment condition. Each data point represents a biological replicate (n = 3) for each treatment condition. **b** Network analysis showing select STAT1- and STAT2-dependent genes identified in differential of differential expression (DDE) analysis (see Fig. 3). 106 DDE genes were identified to have significantly different induction by IFN in Jurkat relative to JLat9.2 cells. Genes are colored according to log2 FC of differential expression of IFN-stimulated genes in JLat9.2 relative to Jurkat cells. Red: greater induction by IFN in JLat9.2 compared to Jurkat cells; Blue: greater induction by IFN in Jurkat cells compared to JLat9.2 cells.

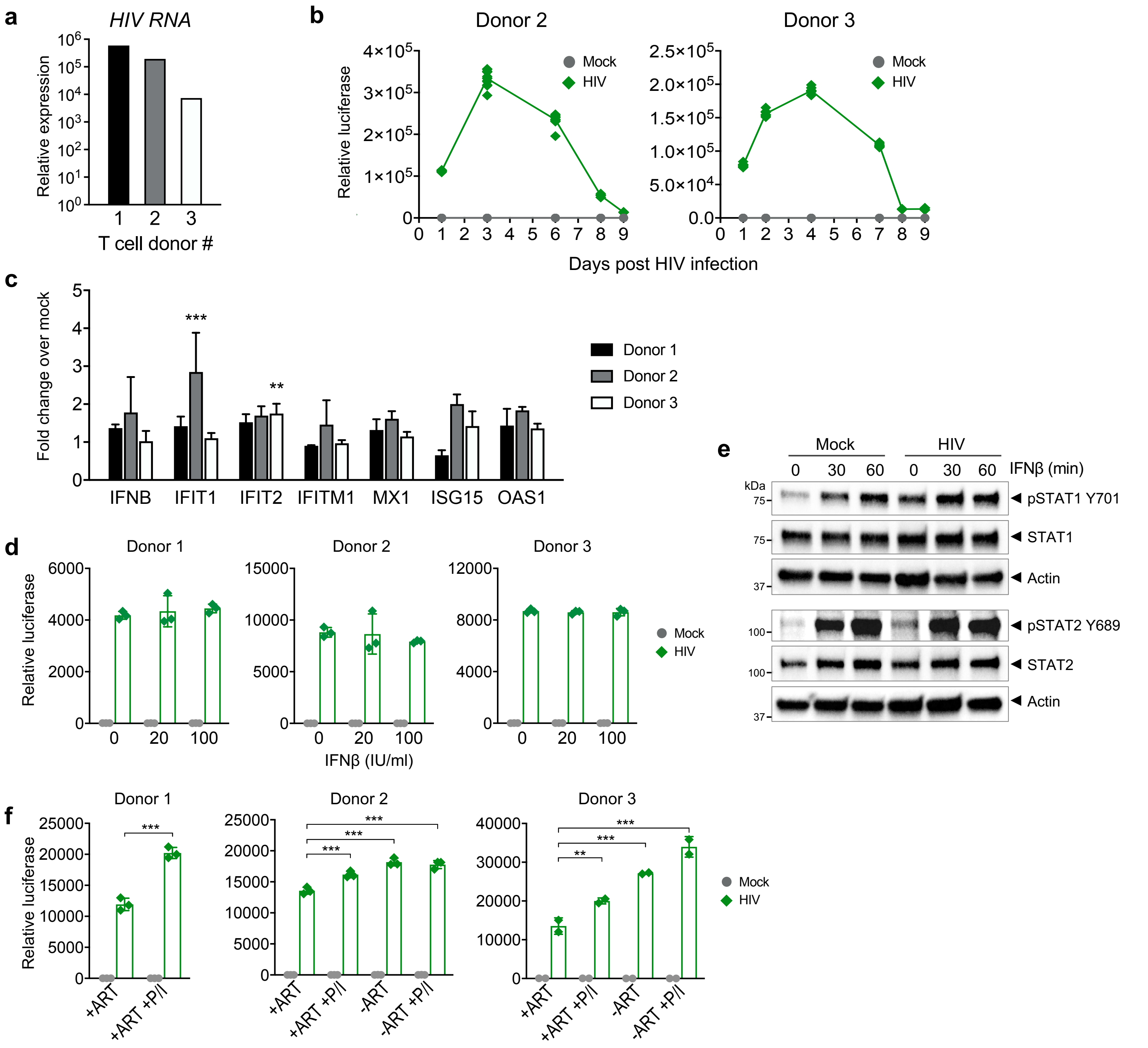

Supplemental Figure 4. Luciferase and ISG expression analysis in a primary CD4+ T cell model of HIV suppression

Primary CD4+ T cells from three healthy human donors were cultured for 5 days in homeostatic cytokines (IL-2, IL-7, IL-15) then mock-infected (media) or infected with NanoLuc HIV at MOI 2.0 (Donor 1 & 2) or MOI 1.0 (Donor 3) for 24h. Viral replication was suppressed for 7 days with ART (10 μm raltegravir and 1 μm efavirenz), then at 8 days post infection (dpi) cells were stimulated with various agonists as detailed below. **a** qRT-PCR analysis of HIV RNA from HIV-infected cells 24h after infection, prior to ART. No HIV RNA was detected in mock-infected samples. Bars represent HIV RNA expression relative to an arbitrary Ct value of 40 from one representative experiment. **b** Supernatant was collected from mock or HIV-infected samples at indicated time points and analyzed for luciferase expression indicating HIV transcription. Data points represent 12 technical replicates collected from each sample in one representative experiment (see also Fig. 5C). **c** qRT-PCR analysis of baseline ISG expression in HIV-infected cells after 7 days of ART suppression (8 days post infection). Bars represent mean FC ± SD relative to mock-infected control for each donor. Data represents three biological replicates per treatment condition. Statistical significance relative to mock-infected control was determined by two-tailed t-test (Holm-Sidak). **d** Luciferase expression analysis of supernatant from mock-infected or HIV-infected samples that were stimulated at 8 dpi with IFNβ (100 IU/ml, 8h). **e** Immunoblot analysis of mock-infected vs HIV-infected CD4+ T cells that were stimulated at 8 dpi with IFNβ (100 IU/ml) for the indicated times (Donor #2 only). **f** Luciferase expression analysis of supernatant from mock-infected or HIV-infected samples that at 8 dpi were cultured with or without ART (24h), and with or without PMA/ionomycin (16nM/1μM, 24h). For Panels d & f, bars represent mean FC + SD luciferase readings of three biological replicates from one experiment for each donor. Statistical significance of IFN-treated relative to untreated cells within each infection group (d) or relative to indicated HIV-infected control (f) was calculated by two-way ANOVA with multiple comparisons (Holm-Sidak). For all data in this figure, multiple independent experiments were performed and data is shown from one representative experiment. For all statistical tests: *p<0.05, **p<0.01, ***p<0.001.

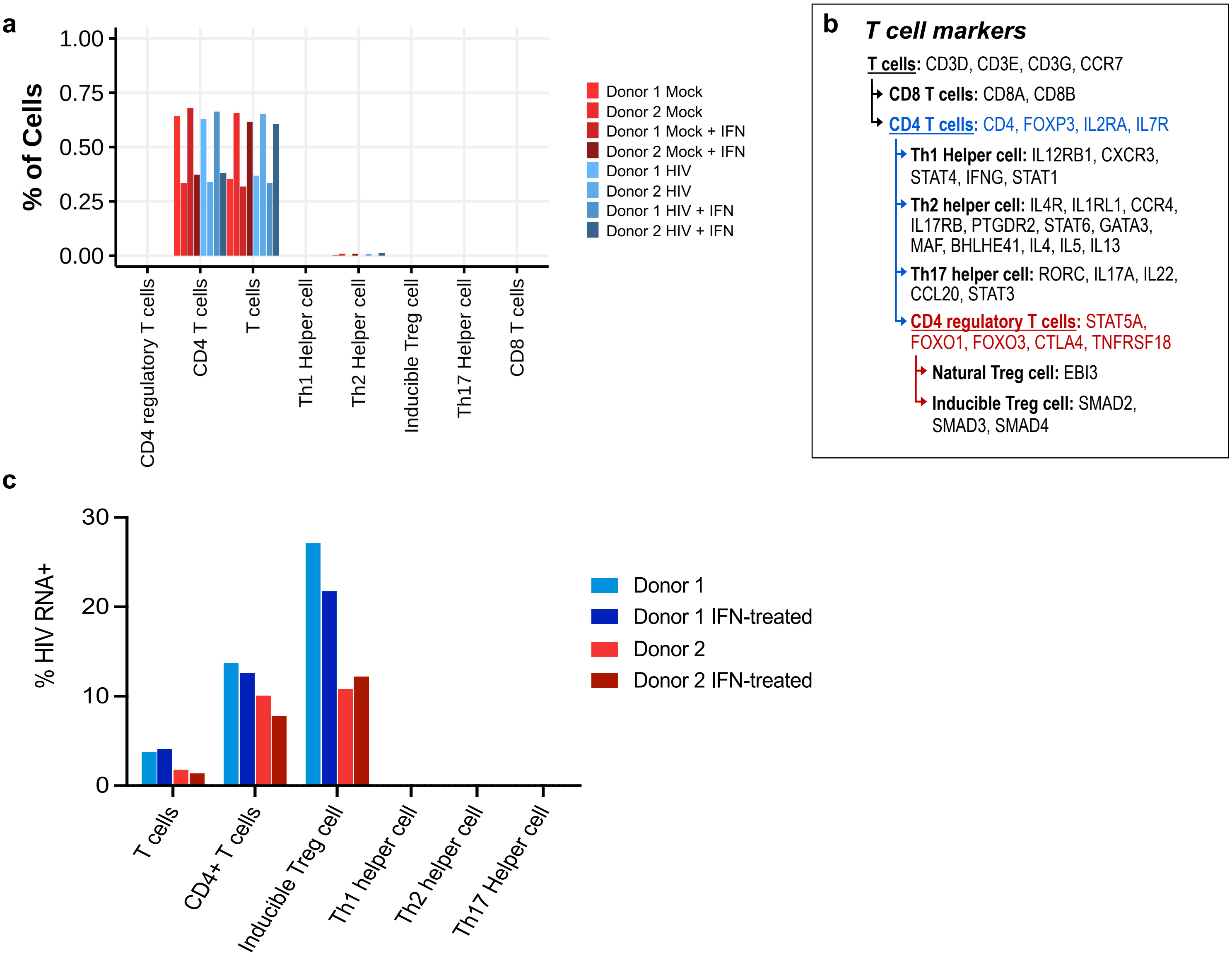

Supplemental Figure 5. scRNA-seq analysis of T cell subsets

Primary CD4+ T cells from two healthy human donors were cultured for 5 days in homeostatic cytokines (IL-2, IL-7, IL-15) then mock-infected (media) or infected with NanoLuc HIV at MOI 2.0 for 24h. Viral replication was then suppressed for 7 days with ART (10 µm raltegravir and 1 µm efavirenz). At day 8 post infection, cells were stimulated for 8h with IFNβ (0, 20, or 100 IU/ml) then analyzed by single cell RNA sequencing (scRNA-seq). **a,b** scRNA-seq analysis of T cell identity per sample using the markers listed on the right**. c** Percent of vRNA+ cells within each T cell subset for each HIV-infected sample.

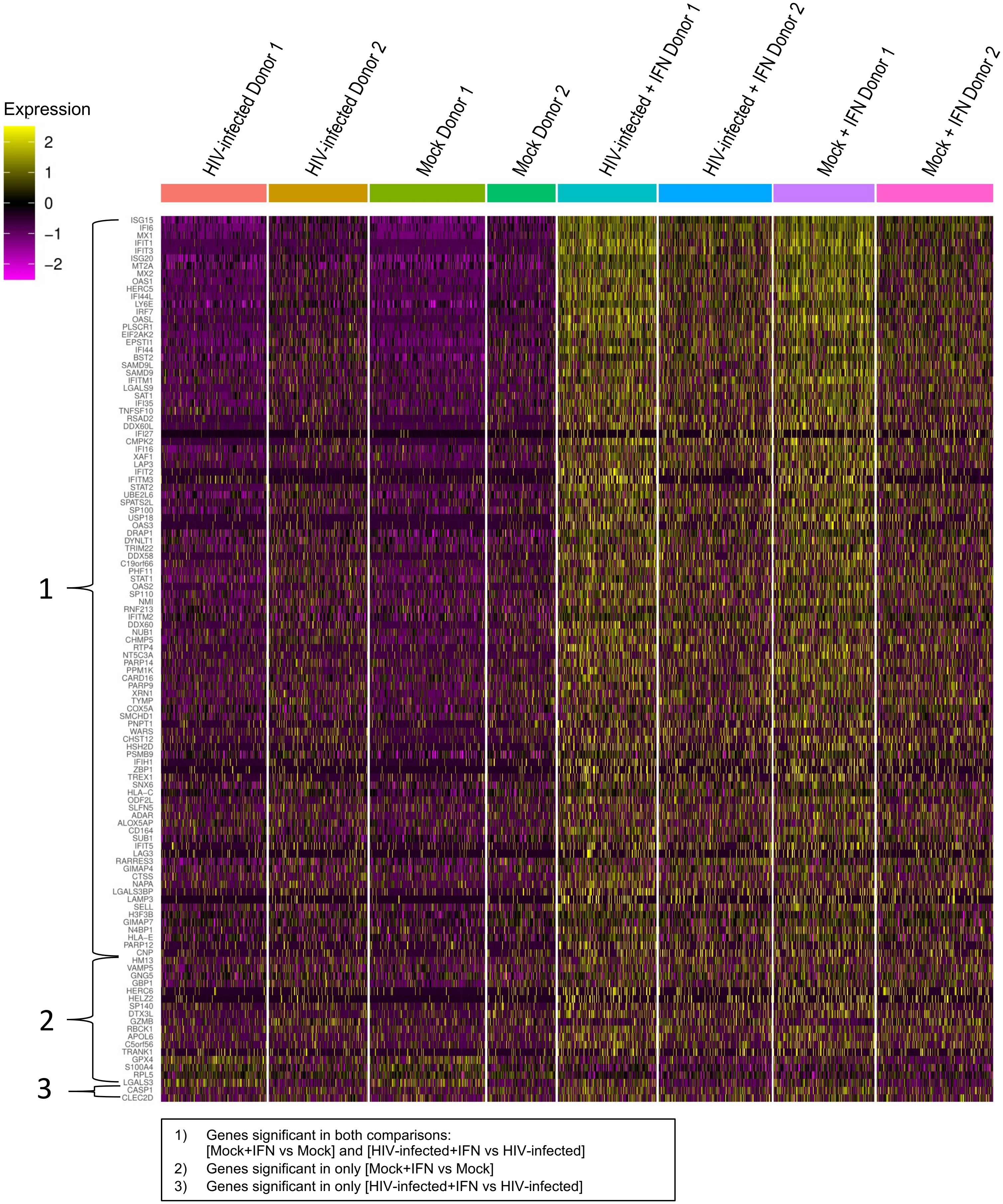

Supplemental Figure 6. scRNA-seq analysis of ISG average expression across all primary CD4+ T cell samples

Heat map showing single cell RNA sequencing (scRNA-seq) analysis of average expression of 116 ISGs in all samples tested. CD4+ T cells from two healthy human donors (Donor 1 & 2) were cultured for 5 days in homeostatic cytokines (IL-2, IL-7, IL-15), then mock-infected (media) or HIV-infected (Nanoluc HIV, MOI 2.0). 24h after infection cells were suppressed with ART for 7 days, then stimulated with IFNβ (100 IU/ml, 8h), and analyzed by scRNA-seq. Each pixel column is the expression of an individual cell. Brackets denote genes significant in each comparison as described in Fig. 7a.

Table S3. HIV expression in primary CD4+ T cells

		% HIV DNA+ (ddPCR)		% HIV RNA+ (sc seq) 8.5 dpi
		24 hpi	8 dpi	
**Donor 1**	Mock	0.002	0.002	0
	Mock + IFN	X	X	0
	HIV infected	29.81	2.47	9.84
	HIV infected + IFN	X	X	9.52
**Donor 2**	Mock	0.008	0.003	0
	Mock + IFN	X	X	0
	HIV infected	24.37	1.54	4.39
	HIV infected + IFN	X	X	3.91
**Donor 3**	Mock	0.24	0.01	X
	Mock + IFN	X	X	X
	HIV infected	63.64	5.24	X
	HIV infected + IFN	X	X	X

X = not tested

Table S4. Oligonucleotides used in this study

Primer ID	Full primer sequence
RT-PCR primers:	
HIV gag fwd	GAAGCTGCAGAATGGGATAGAT
HIV gag rev	GGTTCCTTTGGTCCTTGTCTTA
hIFIT1 fwd	AGAAGCAGGCAATCACAGAAAA
hIFIT1 rev	CTGAAACCGACCATAGTGGAAAT
hIFIT2 fwd	GTTTCCGAAGTGGACATCGCA
hIFIT2 rev	CTGCACAGGTTGTTCTCAGC
hIFITM1 fwd	TACTCCGTGAAGTCTAGGGACAG
hIFITM1 rev	AACAGGATGAATCCAATGGTCA
hIFNB fwd	TGCATTACCTGAAGGCCAAG
hIFNB rev	AAGCAATTGTCCAGTCCA
hISG15 fwd	TGAGCGGGCCCTGAGA
hISG15 rev	ATATCTGGGTGCCTAAGGACCTT
hMX1 fwd	GTTTCCGAAGTGGACATCGCA
hMX1 rev	CTGCACAGGTTGTTCTCAGC
hOAS1 fwd	TGGGTGGTGGAGACCCAA
hOAS1 rev	AATTCAGCCAGGCCTCAGC
hRIGI fwd	GACCCTCCCGGCACAGA
hRIGI rev	TCAGCAACTGAGGTGGCAATC
hRPL13A fwd	GCCCTACGACAAGAAAAAGCG
hRPL13A rev	TACTTCCAGCCAACCTCGTGA
ddPCR primers:	
HIV gag sense	GACTAGCGGAGGCTAGAAGGAGAGA
HIV gag antisense	CTAATTCTCCCCCGCTTAATAYTGACG
